# Immunoprotective Effects of Mori Cortex Radicis Water Extract on Major Aquatic Pathogen (*Aeromonas veronii*) in Crucian Carp

**DOI:** 10.3390/life16060971

**Published:** 2026-06-09

**Authors:** Xing Zhang, Ling Zhu, Yuhang Zhan, Pan Cui, Jing Chen, Shujun Sun, Zijian Ma, Juan Lu, Xiang Liu, Xianjie Liu

**Affiliations:** 1 Anhui Province Key Laboratory of Embryo Development and Reproductive Regulation, Fuyang Normal University, Fuyang 236041, China; 2 Rural Revitalization Collaborative Technology Service Center of Anhui Province, Fuyang Normal University, Fuyang 236041, China; 3 Shenzhen International Institute for Biomedical Research, Shenzhen 518116, China

**Keywords:** Chinese herbal medicine, LC-MS analysis, immune activity, aquatic pathogen, immunologic agent

## Abstract

Mori Cortex Radicis (MCR), which is abundant in resources and low in cost, is a Chinese herbal medicine with antitussive, anti-inflammatory, analgesic, and hypoglycemic effects; however, its application in the prevention and control of aquatic pathogens remains understudied. In this study, a MCR water extract (MCR-WE) was prepared, and its contents of polysaccharides, polyphenols, and proteins were found to be 0.63%, 1.17%, and 2.79%, respectively. LC-MS metabolomics revealed that L(+)-Arginine, 9,12,13-Todea, Citric acid, 1-Deoxynojirimycin, and 4-Guanidinobutanoic acid were the most abundant compounds. Subsequently, by feeding the MCR-WE to crucian carp (*Carassius auratus*) and challenging them with *Aeromonas veronii*, it was found that the MCR-WE enhanced the activities of immune factors (AKP, ACP, LZM) and the phagocytic activity of leukocytes (*p* < 0.05). Furthermore, the MCR-WE improved the survival rate of crucian carp (*p* < 0.05), reduced the bacterial load in the kidneys (*p* < 0.05), decreased the mRNA expression of inflammatory factors (IL-6, IL-1β, TNF-α), and lowered the expression levels of antioxidant-related factors (CAT, GSH-Px, SOD, MDA) and the mRNAs of oxidative stress pathway factors (Nrf2, HO-1, Keap1) (*p* < 0.05). Histopathological sections and immunofluorescence assays showed that the MCR-WE maintained the structural integrity of internal organs and reduced renal cell apoptosis and DNA damage. Therefore, MCR-WE is rich in immunologically active substances, can activate the immune response of crucian carp, reduce fish mortality, exert anti-inflammatory and antioxidant activities, and maintain the structural and functional integrity of internal organs. Thus, the MCR-WE holds promise as a therapeutic agent against *A. veronii* infection in fish.

## 1. Introduction

Mori Cortex Radicis (MCR) is the root cortex of the mulberry tree (*Morus alba* L.), which is widely cultivated in China, offering abundant application resources at low cost. MCR is cold in nature and sweet in taste, with effects of purging the lung, relieving dyspnea, promoting diuresis, and reducing edema. It is commonly used in clinical practice to treat dyspnea with cough, as well as facial and skin swelling [1,2]. Studies have shown that the chemical constituents of MCR mainly include flavonoids and glycosides [3,4], and it has recently gained increasing attention in the field of cosmetic whitening [5]. However, research on the application of MCR for the prevention and control of aquatic pathogens in aquaculture remains insufficient.

Aquaculture provides people with abundant protein-rich food, serves as an important economic pillar, reduces dependence on wild fishery resources, and plays a vital role in food supply, ecological protection, economic development, and employment promotion [6]. With the development of intensive aquaculture technologies, pathogen infections inevitably occur, causing huge economic losses to the aquaculture industry [7,8]. Major pathogenic bacteria in aquaculture include *Aeromonas veronii*, *Aeromonas hydrophila*, *Vibrio alginolyticus*, *Edwardsiella tarda*, and *Vibrio parahaemolyticus* [9,10,11]. *A. veronii* belongs to the genus *Aeromonas* within the family Aeromonadaceae [12]. Its cells are straight rod-shaped to nearly spherical, measuring 0.3–1.0 μm in diameter, and often occur singly, in pairs, or in short chains. It is a Gram-negative opportunistic pathogen widely distributed in aquatic environments and is a major pathogen in aquaculture. It can infect various aquatic animals, including crucian carp, tilapia, giant river prawn (*Macrobrachium rosenbergii*), largemouth bass, Chinese mitten crab, large yellow croaker, and oriental river prawn (*Macrobrachium nipponense*), causing acute hemorrhagic septicemia and chronic ulcerative syndrome [13,14]. It can also infect humans, causing gastroenteritis, septicemia, and other diseases [15].

The prevention and control of *A. veronii* primarily relies on antibiotics, including ceftriaxone, cefotaxime, ciprofloxacin, gentamicin, and amikacin, which inevitably leads to issues such as misuse, bacterial resistance, drug residues, and environmental pollution [16]. Most vaccines against *A. veronii* are still in the laboratory research stage. Studies have found that fish immunized with inactivated or attenuated strains of *A. veronii* confers good immune protective activity [17,18,19]. However, there is a lack of vaccines targeting *A. veronii* in the market. Traditional Chinese medicinal herbs offer advantages such as being green, leaving low residues, and not easily inducing drug resistance in the prevention and control of *Aeromonas* infections. They are mainly classified into those that enhance immunity (e.g., *Astragalus membranaceus*, *Codonopsis pilosula*, *Glycyrrhiza uralensis*) and those that directly combat pathogens (e.g., *Rheum officinale*, *Scutellaria baicalensis*, *Galla chinensis*) [20,21]. Kenanoğlu et al. found that dietary supplementation with extracts of *Urtica dioica* and *Phillyrea latifolia* contributed to a survival rate of 96% in *Sparus aurata* following challenge with *Vibrio anguillarum* [22]. Mukherjee et al. reported that feeding *Ceratophyllum demersum* ethanol extract to *Botia rostrata* enhanced the fish’s resistance against *Aeromonas sobria* [23]. Nasmia et al. demonstrated that palm waste extract exhibited antibacterial activity against major fish pathogens, including *Vibrio harveyi* and *Vibrio parahaemolyticus* [24]. Techaoei conducted a study on the antibacterial activity of water extracts from ten Thai medicinal herbs against fish pathogens, and the results showed that *Caesalpinia sappan* and *Alpinia galangal* possess antibacterial activity against *Aeromonas hydrophila* and *Streptomyces* sp. [25]. However, there is a wide variety of medicinal herbs, and systematic studies on their anti-pathogenic activities are lacking. In particular, further research is needed to identify cost-effective resources for developing novel, low-cost, and highly effective anti-pathogenic agents suitable for the prevention and control of pathogens in aquaculture.

**Figure 1:**
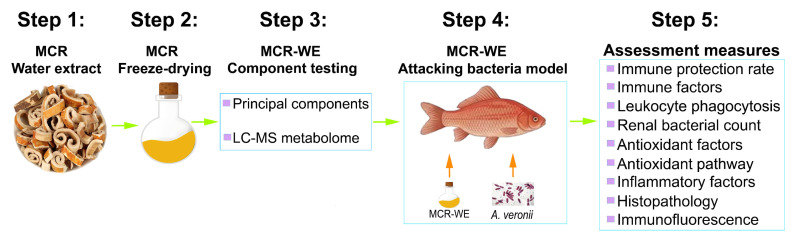
Research approach.

Given that MCR is abundant in resources, low in cost, and may possess immunomodulatory activity, this study prepared a MCR water extract (MCR-WE), and the macromolecular and small-molecular chemical compositions of MCR-WE were analyzed. Then, crucian carp (*Carassius auratus*) were fed with the MCR-WE and subsequently challenged with *A. veronii*. Furthermore, through analyses of immune parameters, leukocyte phagocytic activity, detection of bacterial load in visceral tissues, evaluation of antioxidant and anti-inflammatory activities, assessment of histopathology and cell degeneration and apoptosis of visceral tissues (Figure 1), the immunomodulatory function of the MCR-WE was evaluated, with the aim of developing a novel agent for the prevention and control of aquaculture pathogens.

## 2. Materials and Methods

### 2.1. Strains and Animals

MCR was purchased from Bozhou Kangmei Chinese Herbal Medicine Market (Bozhou, China). *A. veronii* and *Staphylococcus aureus* (*S. aureus*) strains were preserved by the Microbiology Laboratory of Fuyang Normal University. Crucian carp (*C. auratus*, 15 ± 0.5 g) were obtained from Quanzhou Aquaculture Co., Ltd. (Fuyang, China). All animal experiments were conducted in accordance with the Guide for the Care and Use of Laboratory Animals and were approved by the Institutional Animal Care and Use Committee of Fuyang Normal University, China (Approval No. 2025-03-005; 10 March 2025). The zoology experiment and research approach are shown in Figure 1.

### 2.2. Preparation of MCR-WE and Analysis of Polysaccharides, Polyphenols, and Proteins Contents

The MCR-WE was prepared following a previously published method [26]. Briefly, 1 kg of dried MCR was freeze-dried, pulverized using a grinder, and passed through a 60-mesh sieve to obtain a fine powder. The powder was extracted with hot water at a solid-to-liquid ratio of 1:40 (*m*/*v*) at 80 °C for 3 h under continuous stirring at 200 r/min to ensure thorough mixing and consistent heat distribution. The mixture was then centrifuged at 4 °C with 4500 r/min for 20 min to collect the supernatant. The precipitate was subjected to a second hot water extraction under the same conditions. The two supernatants were combined, concentrated by rotary evaporation at 50 °C under reduced pressure (−0.09 MPa) for 8 h, and subsequently freeze-dried to obtain the dried MCR-WE. Furthermore, the contents of polysaccharides, polyphenols, and proteins in the MCR-WE were measured using commercial assay kits (Sangon Biotech Co., Ltd., Shanghai, China), following the manufacturer’s instructions.

### 2.3. LC-MS Metabolomic Analysis of Small Molecule Compounds in MCR-WE

The MCR-WE was analyzed using Liquid Chromatography-Mass Spectrometry (LC-MS) on a UHPLC-Q Exactive system (Thermo Fisher Scientific, Waltham, MA, USA), consisting of ultra-high performance liquid chromatography coupled with Fourier transform mass spectrometry. The separation was performed on an ACQUITY UPLC BEH C18 column (100 mm × 2.1 mm i.d., 1.7 μm) (Waters, Milford, MA, USA). Mobile phase A consisted of 2% acetonitrile in 0.1% formic acid, and mobile phase B consisted of acetonitrile containing 0.1% formic acid. The injection volume was 3 μL, and the column temperature was maintained at 40 °C. The samples were ionized using electrospray ionization, and mass spectra were acquired in both positive (pos) and negative (neg) ion scanning modes. The scan type (*m*/*z*) range was 70–1050. Other parameters were set as follows: heater temperature, 450 °C; capillary temperature, 320 °C; spray voltage (pos), 3500 V; spray voltage (neg), −3000 V; resolution, 70,000. Data were acquired by continuous mass spectrometry scanning, and the total ion current intensity was obtained by summing the intensities of all ions in each mass spectrum. The data were imported into the metabolomics processing software ProgenesisQI v3.0 (Waters, Milford, MA, USA) for baseline filtering, peak detection, integration, retention time correction, and alignment to generate a data matrix containing retention time, mass-to-charge ratio (*m*/*z*), and peak intensity information. The MS and MS/MS mass spectral information was matched against the database of Shanghai Majorbio Bio-pharm Technology Co., Ltd. (Shanghai, China). The mass error for MS was set to less than 10 ppm, and metabolites were identified based on the secondary mass spectrometry match score [27].

### 2.4. Evaluation of the Immunoprotective Rate of MCR-WE

Crucian carp (20 ± 0.5 g) were divided into two groups, with 15 fish in each group. The control group (Ctr) was orally administered 40 μL of sterile water for each fish, while the experimental group was orally administered 40 μL of the MCR-WE (5 mg per fish) (dissolved in sterile water to a concentration of 125 μg/μL) based on a preliminary dose-finding experiment. After being fed once daily for 10 consecutive days, the fish were challenged by intraperitoneal injection with 40 μL of *A. veronii* (1.5 × 10^9^ CFU) based on preliminary experiments. Mortality of the crucian carp was observed for 14 days. The relative percent survival (RPS) was calculated according to the formula: RPS (%) = [1 − mortality rate of MCR-WE (%)/mortality rate of control group (%)] × 100 [28]. Data were analyzed for statistical significance using SPSS 28.0 software.

### 2.5. Detection of Immune Factors

The crucian carp were divided into an MCR-WE group and a control (Ctr) group, with 5 fish in each group. Each fish in the Ctr group was orally administered 40 μL of sterile water, while each fish in the experimental group was immunized with 40 μL of MCR-WE (5 mg per fish). After continuous administration for 10 days, the fish were anesthetized with 40 mg/L MS–222, and serum was collected from the caudal vein. Commercial kits (Shanghai Sangon Biotech Co., Ltd.) were used to detect the levels of acid phosphatase (ACP), alkaline phosphatase (AKP), and lysozyme (LZM) according to the manufacturer’s instructions [29].

### 2.6. Analysis of Leukocyte Phagocytic Function

Crucian carp were divided into two groups (5 fish per group) and orally administered 40 μL of sterile water or MCR-WE (5 mg per fish) for each fish, respectively. After continuous administration for 10 days, the fish were anesthetized, and blood was collected from the caudal vein into an anticoagulant-treated centrifuge tube. Then, 0.2 mL of blood was mixed with 0.2 mL of 1% formaldehyde-inactivated *S. aureus* (1 × 10^8^ CFU). The mixture was incubated in a water bath at 25 °C for 60 min. After incubation, the mixture was dropped onto glass slides and evenly spread to prepare blood smears. The blood smears were stained using a rapid Giemsa staining kit (Sangon Biotech Co., Ltd., Shanghai, China). Following the manufacturer’s instructions, the stained smears were observed under a microscope to count phagocytic cells. The phagocytic percentage (*PP* %) was calculated as: *PP* (%) = (number of phagocytic cells out of 100 counted)/100 × 100%. The phagocytic index (*PI* %) was calculated as: *PI* (%) = (total number of bacteria engulfed by phagocytic cells)/(number of phagocytic cells that participated in phagocytosis) × 100%. Statistical analysis of the data was performed using SPSS 28.0 software [29].

### 2.7. Kidney Bacterial Count

Crucian carp were divided into control and MCR-WE groups (5 fish per group) and orally administered 40 μL of sterile water or MCR-WE (5 mg per fish) for each fish, respectively. After 10 days of continuous administration, the fish were challenged with 40 μL of *A. veronii* (1 × 10^9^ CFU). Three days after bacterial challenge, kidney tissues were collected. Under aseptic conditions, 50 mg of kidney tissue was homogenized with 400 μL of sterile saline using a portable tissue grinder (Shanghai Jingxin Industrial Development Co., Ltd., Shanghai, China). Finally, the kidney homogenate was evenly spread onto the surface of LB medium and incubated overnight in a constant temperature incubator at 37 °C. The number of bacterial colonies on the medium was counted, and the colony count was used to evaluate the clearance effect of MCR-WE against *A. veronii* in crucian carp [29].

### 2.8. Analysis of Antioxidant Factors

Crucian carp were orally administered 40 μL of sterile water or MCR-WE (125 μg/μL), with 5 fish in each group. After 10 days of continuous administration, the fish were challenged with *A. veronii* (1 × 10^9^ CFU). Three days later, serum was collected from the caudal vein of the fish. The levels of catalase (CAT), glutathione peroxidase (GSH-Px), superoxide dismutase (SOD), and malondialdehyde (MDA) were detected using commercial kits according to the manufacturer’s instructions (Nanjing Jiancheng Bioengineering Institute, Nanjing, China). Statistical analysis of the data was performed using SPSS 28.0 software [28].

### 2.9. mRNA Expression Analysis of Inflammatory Factors and Antioxidant Pathway-Related Factors

The mRNA expression levels were evaluated using real-time quantitative PCR (qRT-PCR). Crucian carp were orally administered 40 μL of sterile water or MCR-WE (5 mg per fish), with 9 fish in each group. After 10 days of continuous administration, the fish were challenged with *A. veronii* (1 × 10^9^ CFU). On day 3 post-challenge, the kidneys and spleens were collected, and the kidneys or spleens from three randomly selected fish were pooled together, yielding 3 pooled samples per group. Subsequently, the tissues were mixed with 2 mL of Trizol reagent and immediately homogenized using a portable tissue grinder (Shanghai Jingxin Industrial Development Co., Ltd., Shanghai, China) to prevent RNA degradation. Total RNA was extracted according to the manufacturer’s instructions for the RNA extraction kit (Shanghai Sangon Biotech Co., Ltd., Shanghai, China), and then reverse transcribed into cDNA using a reverse transcription kit (Takara, Beijing, China) following the manufacturer’s instructions. qRT-PCR was performed using the SYBR^®^ Green Premix kit (Takara, Beijing, China) and synthesized primers (Table 1). The ΔCt (cycle threshold change) was obtained by comparing the Ct value of each target gene with that of the internal reference gene (*gapdh*), and then the ΔΔCt was obtained by comparing the ΔCt values between the experimental group and the control group. The mRNA expression levels of inflammatory factors and antioxidant pathway-related factors were analyzed using the 2^−ΔΔCt^ method. Statistical analysis of the data was performed using SPSS 28.0 software [28].

### 2.10. Histopathological Analysis

Crucian carp were orally administered 40 μL of sterile water or MCR-WE (125 μg/μL), with 5 fish in each group. After 10 days of continuous administration, the fish were challenged with *A. veronii* (1 × 10^9^ CFU). Three days later, kidney, spleen, and intestinal tissues were collected and immersed in Davidson’s fixative and 10% formaldehyde solution for 24 h, respectively. After fixation, the tissues were dehydrated through a graded ethanol series (70%, 80%, 90%, 95%, and 100% ethanol), followed by clearing with xylene twice for 30 min. The cleared tissues were embedded in paraffin at 60 °C and sectioned into 4 μm thick slices using a microtome (Leica, Wetzlar, Germany). The sections were placed onto glass slides and dried on a slide dryer at 60 °C for 3 h. Subsequently, the tissue sections were dewaxed with xylene, rehydrated through a graded ethanol series, and cleared with xylene, followed by staining with hematoxylin and eosin (H & E). After staining, the sections were cleared with xylene, mounted with neutral resin, and observed and photographed under an optical microscope [29].

### 2.11. Renal Immunofluorescence Analysis

Renal immunofluorescence analysis was performed according to previously described methods [24]. Briefly, the prepared renal histopathological sections were dewaxed in xylene and then rehydrated in a descending gradient series of ethanol. After antigen retrieval treatment, an immunofluorescence pen was used to mark the boundary around the tissue. Then, 60 μL of 5% bovine serum albumin blocking solution was added into the circle, and the sections were blocked at room temperature for 2 h. After washing with PBST solution, monoclonal antibodies of p53 or γH2A.X (1:500) (Wuhan Triple Eagle Biotechnology Co., Ltd., Wuhan, China) were then added to the tissues, and the sections were incubated overnight at 4 °C. After washing, the secondary antibody solution (1:1000) (Sigma, St. Louis, MO, USA) was added and incubated at 37 °C for 1.5 h. Under room temperature and dark conditions, the cell nuclei were stained with 4′,6-diamidino-2-phenylindole (DAPI) for 10 min, and images were captured under a fluorescence microscope. Fluorescence intensity analysis was performed using ImageJ 1.54g software. Finally, statistical analysis of the data was conducted using SPSS 28.0 software [30].

### 2.12. The Statistical Analysis

All experiments were performed in triplicate. The verification of data normality was assessed by Shapiro–Wilk test. Significant differences between the experimental group and control group were evaluated using SPSS 28.0 software with one-way analysis of variance (ANOVA) followed by Tukey’s multiple comparison test or independent samples *t*-test/Chi-square test. Results were expressed as mean ± standard deviation (SD). *p* < 0.05 was considered statistically significant [29].

## 3. Results

### 3.1. Composition Analysis of MCR-WE

The MCR-WE was analyzed for its major active components using a commercial kit. The results showed that the contents of polysaccharides, polyphenols, and proteins were 0.63%, 1.17%, and 2.79%, respectively (Table 2).

### 3.2. LC-MS Analysis of Small Molecule Compounds in MCR-WE

**Figure 2:**
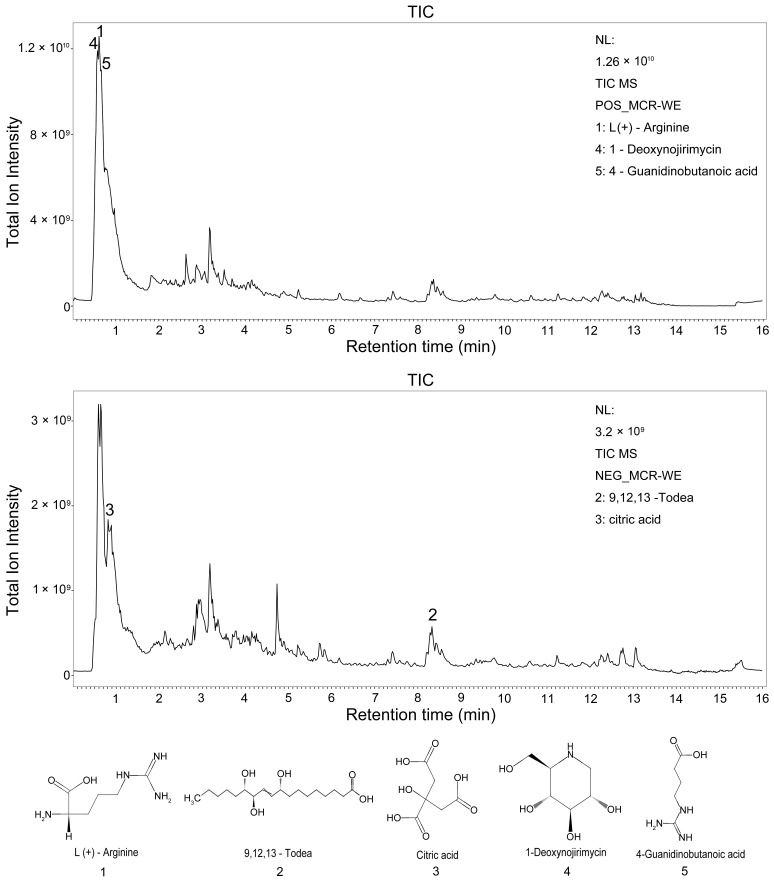
LC-MS metabolomics of MCR-WE.

LC-MS metabolomics was performed to analyze the small molecule compounds in MCR-WE. A total of 645 small molecule compounds were identified (Supplementary Table S1). The results showed that the total ion chromatogram peaks of the samples exhibited good peak shapes and uniform distribution under both positive (pos) and negative (neg) ion modes (Figure 2). The five small molecule compounds with the highest contents were L(+)-Arginine, 9,12,13-Todea, Citric acid, 1-Deoxynojirimycin, and 4-Guanidinobutanoic acid (Table 3).

### 3.3. The Activities of Immune Factors in Crucian Carp Serum

**Figure 3:**
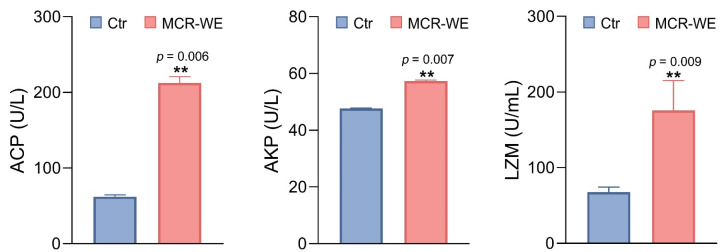
Immune factor activities in crucian carp serum. Statistical significance was determined by independent samples *t*-test. ** *p* < 0.01 (compared with control).

After crucian carp were administered MCR-WE, the activities of immune-related factors (ACP, AKP, LZM) significantly increased in the serum (*p* < 0.05) (Figure 3). This indicates that MCR-WE can activate the non-specific immunity of crucian carp.

### 3.4. Detection of Phagocytic Capacity of Leukocytes in Crucian Carp Blood

After crucian carp were administered MCR-WE for 10 days, blood was collected to detect leukocyte phagocytic activity. The results showed that compared with the control group, both the phagocytic index (*PI* %) and phagocytic percentage (*PP* %) of crucian carp leukocytes were significantly increased in the MCR-WE group (*p* < 0.05) (Table 4).

### 3.5. Immune Protection Rate of MCR-WE Against A. veronii

**Figure 4:**
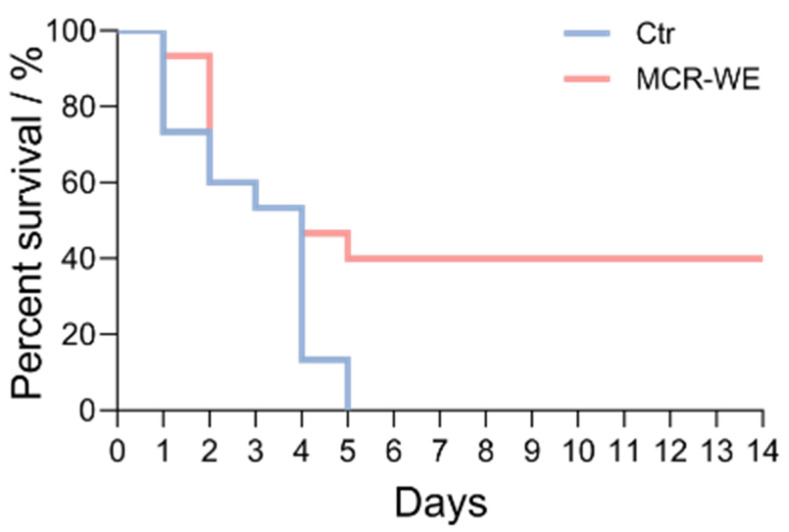
Survival rate of crucian carp fed with MCR-WE and challenged with *A. veronii*.

Crucian carp were fed with MCR-WE and then challenged with *A. veronii*. The results showed that crucian carp exhibited sluggish movement and reduced feeding in both the control group and the MCR-WE group. In the control group, all fish died within 5 days, while mortality in the MCR-WE group stabilized after 5 days (Figure 4). The immune protection rate of MCR-WE was 40% (*p* < 0.01) (Table 5). These findings indicate that MCR-WE enhances the ability of crucian carp to resist *A. veronii* infection.

### 3.6. Kidney Bacterial Count in Crucian Carp

**Figure 5:**
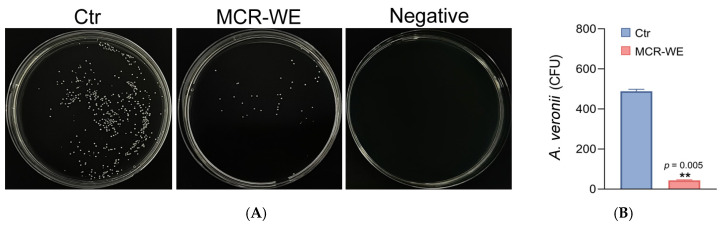
Bacterial load in the kidneys of crucian carp. (**A**,**B**) represent the bacterial spread plates and bacterial count, respectively. Negative: kidney without bacterial infection served as a negative control. Statistical significance was determined by independent samples *t*-test. ** *p* < 0.01 (compared with control).

Crucian carp were fed with MCR-WE and then challenged with *A. veronii*. Three days after the challenge, the kidneys were collected for bacterial count experiments. Compared with the control group, the bacterial count in the kidneys of crucian carp was significantly reduced in the MCR-WE group (*p* < 0.01) (Figure 5). This indicates that MCR-WE can reduce *A. veronii* infection in the kidney of crucian carp.

### 3.7. Analysis of Serum Antioxidant Factors in Crucian Carp

**Figure 6:**
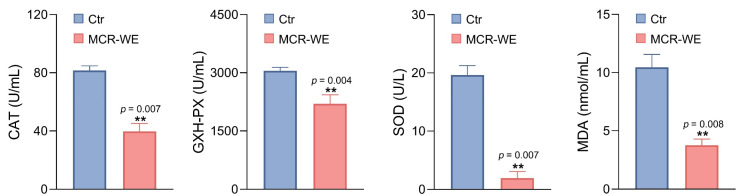
Analysis of antioxidant-related factors in the serum of crucian carp. Statistical significance was determined by independent samples *t*-test. ** *p* < 0.01 (compared with control).

After being fed MCR-WE continuously for 10 days, crucian carp were challenged with *A. veronii*. Three days post-infection, serum samples were collected from fish for antioxidant factor analysis. Compared with the control group, the levels of antioxidant-related factors (CAT, GSH-PX, SOD, and MDA) were significantly decreased (*p* < 0.01) in the serum of MCR-WE group (Figure 6). These findings indicate that crucian carp fed with MCR-WE exhibited a lower oxidative stress response, suggesting that MCR-WE possesses antioxidant properties.

### 3.8. mRNA Expression of Key Factors Related to Antioxidant Metabolic Pathways

**Figure 7:**
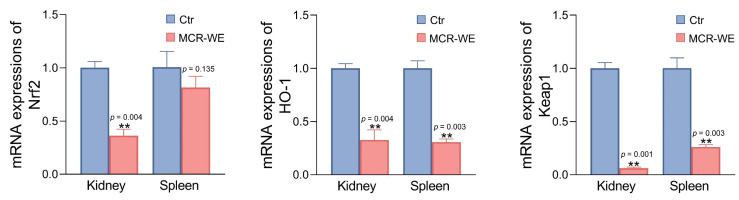
mRNA expression of key factors in the antioxidant metabolic pathway. Statistical significance was determined by independent samples *t*-test. ** *p* < 0.01 (compared with control).

On the third day after crucian carp were fed with MCR-WE and were challenged with *A. veronii*, kidney and spleen tissues were collected for qRT-PCR analysis. The results showed that the mRNA expression levels of key factors (Nrf2, HO-1, Keap1) related to antioxidant pathway were significantly decreased (*p* < 0.05) (Figure 7). These findings indicate that the level of antioxidative stress was reduced in crucian carp, suggesting that MCR-WE possesses antioxidant properties.

### 3.9. mRNA Expression of Inflammatory Factors

**Figure 8:**
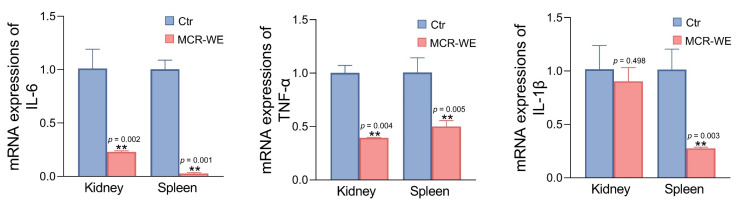
mRNA expression of inflammation-related factors. Statistical significance was determined by independent samples *t*-test. ** *p* < 0.01 (compared with control).

Crucian carp were fed with MCR-WE and then challenged with *A. veronii*. Three days later, total RNA was extracted from tissues (kidney and spleen), and the mRNA expression of inflammatory factors (IL-6, TNF-α, and IL-1β) were detected. The results indicated that the mRNA expression levels of most inflammatory factors exhibited a decreasing trend (*p* < 0.05) (Figure 8). These findings suggest that MCR-WE can reduce the inflammatory response in crucian carp during *A. veronii* infection, demonstrating that MCR-WE possesses anti-inflammatory effects.

### 3.10. Histopathological Observation of Crucian Carp Tissues

**Figure 9:**
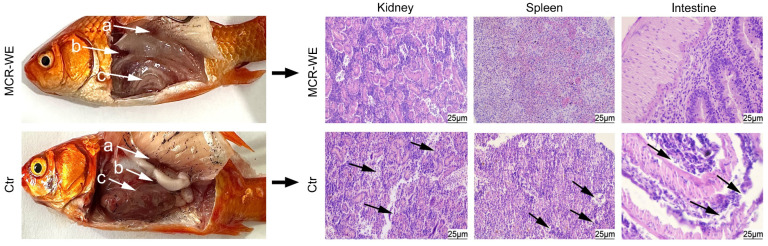
Histopathological examination of the kidney, spleen, and intestinal tissues of crucian carp. a, b, c represents the kidney, spleen, and intestine, respectively.

Crucian carp were fed with MCR-WE for 10 days and then challenged with *A. veronii*. Three days post-infection, the internal organs (kidney, spleen, and intestine) of crucian carp were collected for histopathological section observation. The results showed that in the control group, the kidney tissue exhibited a loose structure, with degeneration and apoptosis of renal tubules and glomerular cells; the spleen showed decreased cell density and increased intercellular space; the intestinal mucosal layer displayed incomplete cell structure with cell degeneration and apoptosis. In the MCR-WE group, the structures of the kidney, spleen, and intestine remained intact, with no observed cell degeneration or apoptosis (Figure 9).

### 3.11. Immunofluorescence Analysis of the Kidney

**Figure 10:**
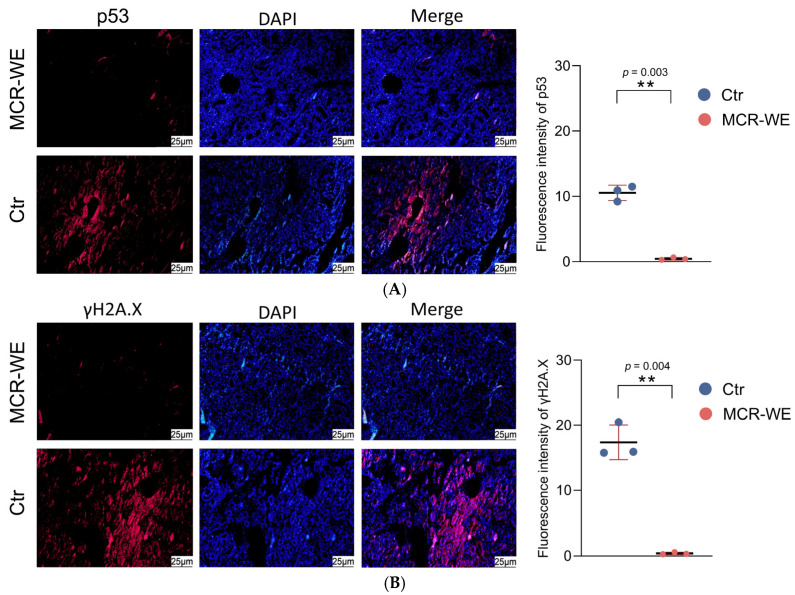
Immunofluorescence detection of p53 (**A**) and γH2A.X (**B**) in the kidney tissue of crucian carp. Statistical significance was determined by independent samples *t*-test. ** *p* < 0.01 (compared with control group).

Crucian carp were fed with MCR-WE and then challenged with *A. veronii*. Three days later, kidney tissues of crucian carp were collected for immunofluorescence analysis. Blue fluorescence indicates stained cell nuclei, while red fluorescence represents the fluorescence intensity of p53 and γH2A.X proteins. The fluorescence intensity reflects the expression levels of these two proteins. The results showed that the fluorescence intensity of p53 and γH2A.X in the MCR-WE group was significantly lower than that of the control group (*p* < 0.05) (Figure 10). These findings indicate that MCR-WE can protect against cell apoptosis and DNA damage in internal organs induced by bacterial infection.

## 4. Discussion

MCR is a traditional Chinese herbal medicine renowned for its antitussive, anti-inflammatory, analgesic, and hypoglycemic effects, and its functional properties have attracted research attention. Studies have shown that the main components of MCR include flavonoids, coumarins, polysaccharides, tannins, and volatile oils [31]. Wang et al. identified four new benzofuran-type stilbene glycosides from MCR, along with 14 known compounds, including eight benzofuran-type stilbenes and six flavonoids [32]. Using high-performance liquid chromatography (HPLC) coupled with a photodiode array, Seo et al. analyzed five components (neochlorogenic acid, chlorogenic acid, cryptochlorogenic acid, caffeic acid, and coumaric acid) from MCR and found that the extract exhibited anti-inflammatory effects [31]. He et al., using high-performance affinity chromatography coupled with ESI-MS, discovered that morin from MCR can bind to the beta1-adrenergic receptor and beta2-adrenergic receptor, while mulberroside C binds to the β2-AR, suggesting that mulberroside may be involved in stress response [33]. A water extract refers to a concentrated product obtained by extracting active components from plants using water as a solvent through physical methods (such as decoction, maceration, reflux, or percolation). The core principle relies on the solubility of water for polar substances [34]. Zeng et al. administered a water extract of MCR to mice and found that the water extract inhibited cigarette smoke-induced aging of lung tissue by targeting the PI3K/Akt signaling pathway [35]. In this study, a water extract of MCR was obtained, and its contents of polysaccharides, polyphenols, and proteins were determined to be 0.63%, 1.17%, and 2.79%, respectively. Using LC-MS technology to analyze the small-molecule substances in the MCR-WE, it was found that L (+)-Arginine, 9,12,13-Todea, Citric acid, 1-Deoxynojirimycin, and 4-Guanidinobutanoic acid had the highest contents. Since the temperature (50 °C) during extraction and rotary evaporation concentration of the MCR-WE is relatively high, some volatile or highly thermolabile constituents may be partially lost, and this represents a limitation of the aqueous extraction approach. Polyphenols, polysaccharides, proteins, and these small-molecule substances are closely related to immune function. However, in previous studies, research on the detection of macromolecular substances in MCR-WE, especially the analysis of small molecular substances, was insufficient. This study fills this gap, and lays the foundation for its subsequent application in aquaculture.

MCR extract possesses immunomodulatory properties. Its polysaccharide components activate macrophages and natural killer cells to enhance their phagocytic and cytotoxic activities, thereby improving the host’s ability to clear pathogens [36]. Furthermore, MCR extract significantly regulates serum inflammatory cytokine levels and the proportion of T lymphocyte subsets, suggesting its immunomodulatory potential in chronic inflammatory airway diseases [31]. In this study, crucian carp were orally administered MCR-WE and subsequently challenged with pathogenic bacteria. The results showed a reduction in *A. veronii* load in kidney tissue and a significant decrease in mortality in the MCR-WE group, indicating that the extract enhances the body’s ability to clear bacteria. Additionally, the MCR-WE administration group increased the activity of immune factors (AKP, ACP, LZM), enhanced leukocyte phagocytic activity, and the immunoprotection rate against *A. veronii* infection was 40% in fish. Previous studies have shown that immunization with extracts from *Urtica dioica*, *Ceratophyllum demersum*, *Caesalpinia sappan*, and *Alpinia galangal* provides immune protection rates against bacterial infection in fish of approximately 30–60% [22,23,25], which is close to the 40% observed in the present study. These extracts activate fish immune factors, which is consistent with the results of this study. Additionally, this study further revealed that after immunization with MCR-WE, the phagocytic activity of fish leukocytes was enhanced, and the ability of visceral tissues to clear bacteria was increased. Therefore, MCR-WE administration may enhance the resistance of crucian carp to bacterial infection by activating non-specific immunity.

In this study, the administration dosage of MCR-WE (5 mg per fish, ~250 mg/kg body weight) was determined based on a preliminary dose-finding experiment in which three dosages (2, 5, and 8 mg per fish) were evaluated for their effects on immune factors activity (AKP and ACP) and survival following *A. veronii* challenge. The 5 mg dose produced the most consistent and significant immunostimulatory effects without observable adverse effects on fish behavior, feed intake, or growth. The 8 mg dose showed no additional benefit and caused mild mucus hypersecretion in a subset of fish. This dosage is also within the range reported for other plant extracts used as immunostimulants in fish (typically 200–500 mg/kg body weight per day) [23,25,37]. Moreover, the 10-day oral administration period in this research was selected based on reported lag times for herbally induced immune priming in fish. Studies on similar plant extract immunostimulants (e.g., *Astragalus polysaccharides*, *Agaricus bisporus*) indicate that 7–14 days of pre-treatment is required to achieve significant elevation of serum immune factor activities [24,37]. A 10-day administration period of MCR-WE was chosen as a practical compromise between efficacy and feasibility for aquaculture application.

Studies have shown that MCR can regulate the release of factors related to anti-inflammatory pathways. Its flavonoid compounds (morusin) inhibit the release of inflammatory cytokines, thereby reducing tissue damage; simultaneously, they scavenge free radicals, alleviate oxidative stress, and protect immune cell function [38]. Xiao et al. found that after immunizing fish with the protein, DNA, and IgY antibody vaccines of *Vibrio fluvialis* VF08100 protein and subsequently challenging them with different pathogenic bacteria, the expression of antioxidant-related factors (MDA, GSH-Px, CAT, SOD) and inflammatory cytokine mRNAs (IL-6, IL-8, IL-1β, TNF-α) was reduced, thereby decreasing oxidative stress and inflammatory responses [28]. This study found that after crucian carp were orally administered with MCR-WE and challenged with *A. veronii*, the expression of antioxidant-related factors and inflammatory cytokine mRNAs were reduced. Thus, the MCR-WE possesses the ability to reduce oxidative stress and inflammatory responses induced by bacterial infection. The Nrf2/HO-1 pathway is an important intracellular anti-oxidative stress pathway [39]. Nrf2 is a nuclear transcription factor that induces the expression of various antioxidant enzymes, scavenges intracellular free radicals and oxidative substances, and protects cells against oxidative stress-induced damage [40,41]. HO-1 is heme oxygenase, which catalyzes the degradation of heme into biliverdin, carbon monoxide, and iron, and these products possess antioxidant effects [42]. Keap1 is a protein that plays a regulatory role in cellular oxidative stress responses. Keap1 maintains intracellular redox homeostasis by binding to Nrf2 and promoting its ubiquitination and degradation [43]. This study showed that after crucian carp were immunized with MCR-WE and challenged with *A. veronii*, the mRNA expression of Nrf2, HO-1, and Keap1 was significantly reduced, indicating the activity of the oxidative stress pathway decreased and a weakened oxidative stress response in the crucian carp. Addition, the MCR-WE administration group showed intact renal, splenic, and intestinal tissues, with reduced p53 and γH2A.X fluorescence signals, indicating less cellular damage, and the results were consistent with reduced oxidative stress rather than impaired antioxidant defense. Thus, after fish were immunized with MCR-WE and challenged with bacteria, the reduced damage in the animal body resulted in attenuated inflammatory responses and oxidative stress, downregulation of the Nrf2/Keap1/HO-1 antioxidant pathway, and consequently decreased expression of anti-inflammatory and antioxidant-related factors. Overall, the MCR-WE administration reduces oxidative stress and inflammatory responses induced by bacterial infection in crucian carp.

The integrity of internal organ tissue structure is essential for maintaining the physiological functions of animals, and differences in changes to the internal organ tissue structure can be used to assess the immunological functions of drugs [44]. Chen et al. immunized fish with IgY antibodies of live or inactivated *A. veronii* and subsequently challenged fish with *A. veronii* and *A. hydrophila*. Histopathological sections revealed that the internal organ structures of the experimental group remained intact [30]. Cui et al. immunized fish with IgY derived from live or inactivated *Pseudomonas anguilliseptica* and challenged them with *A. veronii* and *P. anguilliseptica*. Using histopathological sections, they also found that IgY antibodies protected the integrity of internal organ tissues [29]. In this study, crucian carp were immunized with MCR-WE and subsequently challenged with pathogenic bacteria. Compared to the control group, the MCR-WE group exhibited more intact histopathological structures in the kidney, spleen, and intestine. It is evident that the MCR-WE improved the integrity of the internal organ tissues of fish following bacterial infection. Furthermore, the p53 protein maintains genomic stability by regulating the cell cycle and apoptosis, and serves as an important indicator of apoptosis [45]. γH2A.X is a phosphorylated form of the Histone H2A.X protein. It recruits DNA repair proteins to sites of DNA damage to maintain genomic integrity and serves as an indicator of DNA damage [46,47]. In this study, it was found that after immunizing fish with the MCR-WE and challenging them with *A. veronii*, the immunofluorescence signals of p53 and γH2A.X in the kidney were significantly reduced, indicating decreased expression levels of p53 and γH2A.X. Previous studies have shown that immunizing fish with active pharmaceutical agents reduces bacterial damage to the body by activating the animal’s immune response, thereby maintaining the structural and functional integrity of visceral tissues [29,30]. This is consistent with the findings of the pathological experiments in the present study, which revealed that MCR-WE administration preserves visceral structural integrity. Furthermore, this study also found decreased expression of p53 and γH2A.X, two key pathway proteins involved in cell apoptosis. Thus, immunization with MCR-WE in fish can downregulate the apoptotic pathway induced by bacterial infection, thereby maintaining the structural integrity of visceral tissues. Therefore, MCR-WE possesses immunostimulatory activity.

## 5. Conclusions

In this study, an MCR-WE was prepared, and the contents of both macromolecular and small molecular substances in the extract were measured. Subsequently, using a model in which crucian carp were fed the MCR-WE and challenged with *A. veronii*, it was found that the MCR-WE administration enhances the immune protection of crucian carp against *A. veronii* by activating the non-specific immunity. Moreover, follow-up immunoprotective experiments with a larger number of fish need to be conducted in aquaculture settings. In future studies, investigations into the immune activation effect of MCR-WE on different fish species, as well as its protective efficacy against various aquaculture bacterial and viral pathogens, need to be conducted. Additionally, dose optimization and pharmacokinetic analysis of MCR in fish, along with safety evaluation of long-term feeding, also require further analysis. In summary, this study expanded the new uses of MCR, and given the abundant resources and low cost of MCR, it has potential as a pharmaceutical agent for the prevention and treatment of *A. veronii* infection in fish.

## Figures and Tables

**Table 1:** qRT-PCR primers for the mRNA expression evaluation.

Proteins	NCBI Accession Number	Forward Primer (5′-3′)	Reverse Primer (5′-3′)
GAPDH	XM_026284269.1	GATTTCAACGGGGATGTGCG	TCACACACACGGTTGCTGTA
IL-6	XM_026289280.1	TCTCCTCAGACCCTCAGACG	CGTTTGGTCCCGTGTTTGAC
TNF-α	EU069817.1	GGGCCACATCGTGATTCGTA	GCCTCCAGTGTAGCATGTGT
IL-1β	AJ249136.1	TTCAGGAAAGAGACGGGCAC	GTCAGTTGGCACCTGGATCA
Nrf2	XM_042763189.1	ATCTGCACAGCTTCTCACCT	AGTCAGAGTCGCTGAAACCA
HO-1	KC758864.1	CAGCTCTCCAGTGCTACAGT	TCAGCCTGTGGATTTGACCT
Keap1	XP_026101140.1	ATGTACCAGATCGACAGCGT	TTGAAGAACTCCTCCTGCGT

**Table 2:** Analysis of the major components of MCR-WE.

NO.	Component	Content Ratio (%)
1	Polysaccharides	0.63 ± 0.02
2	Polyphenols	1.17 ± 0.02
3	Proteins	2.79 ± 0.03

**Table 3:** LC-MS metabolomics data of the five most abundant small molecule compounds.

NO.	Metabolite	Formula	M/Z	Retention Time/min	Fragmentation Score	Mode	Abundance
1	L(+)-Arginine	C6H14N4O2	175.1188	0.5607	93.2	pos	60904829
2	9,12,13-Todea	C18H34O5	329.233	8.3127	91.3	neg	11811272
3	Citric acid	C6H8O7	191.0189	0.8365	97.6	neg	11529805
4	1-Deoxynojirimycin	C6H13NO4	164.0916	0.5252	96	pos	11187494
5	4-Guanidinobutanoic acid	C5H11N3O2	146.0922	0.6321	94.7	pos	9568950

**Table 4:** Phagocytic activity analysis of leukocytes in crucian carp.

Group	Phagocytic Percentage (*PP* %)	Phagocytic Index (*PI* %)	*PP* % (*p*-Value)	*PI* % (*p*-Value)
Control	20 ± 6.67	44.44 ± 9.62	--	--
MCR-WE	55.56 ± 15.4 *	139.44 ± 8.27 **	0.021	0.003

Statistical significance was determined by independent samples *t*-test. * *p* < 0.05, ** *p* < 0.01 (compared with control).

**Table 5:** Immune protection rate of MCR-WE in crucian carp.

Group	Total, No.	Survived, No.	Death, No.	ADR, %	RPS, %	RPS, *p*-Value
Control	15	0	15	100	--	--
MCR-WE	15	6	9	60	40 **	0.006

ADR, accumulating death rate. RPS, relative percentage survival. RPS (%) = [1 − mortality rate of MCR-WE (%)/mortality rate of control group (%)] × 100. Control represents the fish feed sterile water without MCR-WE. Statistical significance was determined by Chi-square test. ** *p* < 0.01 (compared with control).

## Data Availability

The data presented in this study are available on request from the corresponding author.

## Supplementary Materials

The following supporting information can be downloaded at: https://www.mdpi.com/article/10.3390/life16060971/s1, Table S1: LC-MS analysis of small molecule compounds in MCR-WE.
