# Supplementary material for: Immunoprotective Effects of Mori Cortex Radicis Water Extract on Major Aquatic Pathogen (Aeromonas veronii) in Crucian Carp
Source: Life (Basel). 2026 Jun 9;16(6):971. doi: 10.3390/life16060971 (PMC13301265; doi:10.3390/life16060971)
Supplement: Supplementary file 1 [file life-16-00971-s001.zip › Supplementary Table S1.pdf]

Supplementary Table S1. LC-MS analysis of small molecule compounds in MCR-WE.

NO.	Metabolite	Formula	M/Z	Retention time	Fragmentation Score	Mode	Abundance
1	L(+)-Arginine	C6H14N4O2	175.1188	0.5607	93.2	pos	60904829
2	9,12,13-Todea	C18H34O5	329.233	8.3127	91.3	neg	11811272
3	Citric acid	C6H8O7	191.0189	0.8365	97.6	neg	11529805
4	1-Deoxynojirimycin	C6H13NO4	164.0916	0.5252	96	pos	11187494
5	4-Guanidinobutanoic acid	C5H11N3O2	146.0922	0.6321	94.7	pos	9568950
6	Kelampayoside A	C20H30O13	477.1608	3.1636	79.5	neg	7830540
7	D-(-)-Quinic acid	C7H12O6	191.055	0.6086	79.4	neg	5474832
8	Chlorogenic Acid	C16H18O9	353.0875	2.8973	97.1	neg	4813197
9	Malic acid	C4H6O5	133.013	0.6268	99.2	neg	4802628
10	Antiarol	C9H12O4	185.0807	3.1739	77.3	pos	4608886
11	Octadec-9-ene-1,18-dioic-acid	C18H32O4	311.2225	12.411	86.8	neg	3990387
12	9-Oxo-10,12-octadecadienoic acid	C18H30O3	295.2262	8.362	96.4	pos	3952452
13	2-Pyrrolidinecarboxylic acid	C5H9NO2	116.0708	0.6144	92.9	pos	3844620
14	Isocitrate	C6H8O7	191.0189	0.6626	66.1	neg	3644381
15	D-(+)-Lactose	C12H22O11	341.1085	0.6086	91.9	neg	3367865
16	Nonanedioic acid	C9H16O4	187.0967	4.7268	98.8	neg	2817302
17	Deoxyandrographolide	C20H30O4	335.2186	11.2549	37.5	pos	2471306
18	9(S)-HOTrE	C18H30O3	295.2263	12.2675	90.8	pos	2378035
19	Gorlic acid	C18H30O2	279.2314	13.0367	92.9	pos	2269348
20	5-Hydroxycoumarin	C9H6O3	163.0388	2.9288	93.7	pos	2238513
21	Guanine	C5H5N5O	152.0566	0.9699	87.4	pos	2220662
22	9(s)-hode	C18H32O3	295.2274	13.055	95.1	neg	2190085
23	4-Hydroxyisoleucine	C6H13NO3	148.0966	0.5607	51.2	pos	2130890
24	L-nmma	C7H16N4O2	189.1343	0.5788	76.7	pos	2050626
25	2-Isopropylmalic acid	C7H12O5	175.0602	2.9602	98	neg	2001919
26	Tianshic acid	C18H34O5	295.2263	10.7455	78.9	pos	1928020
27	Gluconic acid	C6H12O7	195.0501	0.5729	93	neg	1829496
28	Caffeic acid	C9H8O4	179.0341	3.1446	98.4	neg	1573185
29	3-(4-Hydroxy-3-methoxyphenyl)propanal	C10H12O3	181.0858	3.5138	83.7	pos	1562001
30	4-p-Coumaroylquinic acid	C16H18O8	337.0928	3.3701	92.4	neg	1464118
31	Stearidonic acid	C18H28O2	277.2157	8.362	85.6	pos	1456251
32	5,7,3',4',5'-pentahydroxyflavanone	C15H12O7	303.0508	4.2025	36.3	neg	1455315
33	1,2,3-Trihydroxybenzene	C6H6O3	127.0391	0.6144	76.3	pos	1433745
34	13-OxoODE	C18H30O3	295.2262	13.1696	96	pos	1369060
35	Scopolin	C16H18O9	355.1017	2.9288	64.3	pos	1368077
36	Indoline	C8H9N	120.081	1.8598	62.4	pos	1319908
37	Rosiridoside B	C21H36O11	463.2184	3.7239	80.2	neg	1187348
38	Adenosine	C10H13N5O4	268.1036	0.9699	89.1	pos	1182766
39	Sarracenin	C11H14O5	227.091	3.1739	50.7	pos	1158859
40	Isoguanosine	C10H13N5O5	284.0985	0.9699	83.4	pos	1144848
41	4-(beta-D-glucosyloxy)-3-hydroxy-benzoic acid	C13H16O9	315.0721	2.0501	75.2	neg	1084435
42	Sebacic acid	C10H18O4	201.1125	5.6924	97.6	neg	1030962

43	17-Hydroxylinolenic acid	C18H30O3	295.2262	11.8281	95	pos	944010
44	Viscidulin I	C15H10O7	301.0352	5.0401	76.1	neg	937384
45	Orcinol glucoside	C13H18O7	287.112	2.8742	57.6	pos	933693
46	Alpha-D-Glucose	C6H12O6	179.0551	0.5907	70.1	neg	927083
47	Nandrolone	C18H26O2	275.2002	11.0771	73.7	pos	863830
48	1-O-Caffeoylquinic acid	C16H18O9	707.182	2.8973	94.7	neg	862392
49	Cis-2,8-Menthadien-1-ol	C10H16O	153.1273	3.7338	56.5	pos	761477
50	Isoscopoletin	C10H8O4	193.0494	4.056	96.4	pos	706481
51	(+)-Muscarine	C9H19NO2	174.1488	3.0982	47.6	pos	692676
52	4-aminobutanoic acid	C4H9NO2	104.0711	0.5788	79.4	pos	681254
53	4-Caffeoylquinic acid	C16H18O9	353.0877	2.4524	91.6	neg	676050
54	4-Hydroxy-2-methylacetophenone	C9H10O2	151.0753	3.2839	88.7	pos	674991
55	Coronaric acid	C18H32O3	297.2419	12.289	69.7	pos	649033
56	Scopoletin	C10H8O4	193.0494	3.0792	97.9	pos	637084
57	Carnosic acid	C20H28O4	333.203	12.7934	35.2	pos	636804
58	2,6-Dimethoxyphenol	C8H10O3	155.0701	2.8742	73.8	pos	622554
59	Phaseoloidin	C14H18O9	329.0876	2.1337	63.7	neg	590142
60	Betaine	C5H11NO2	118.0864	0.5966	82.1	pos	588240
61	Tuberonic acid	C12H18O4	225.1127	6.1583	94	neg	560040
62	Cytosine	C4H5N3O	112.0509	0.6144	37.9	pos	550022
63	Mesaconic acid	C5H6O4	129.0181	0.8745	70.5	neg	529904
64	Citrinin	C13H14O5	251.091	2.8742	70.7	pos	521252
65	Lactobionic acid	C12H22O12	357.1035	0.5729	58.4	neg	491734
66	Aromatic aldehyde	C7H6O	107.0496	1.9558	94.7	pos	489132
67	Succinic acid	C4H6O4	117.0181	0.9712	64.7	neg	488117
68	4-Coumaric acid	C9H8O3	163.039	3.7653	90.8	neg	484724
69	Coumarin	C9H6O2	147.044	3.3586	96	pos	476359
70	(R)-(+)-2-Pyrrolidone-5-carboxylic acid	C5H7NO3	130.05	0.8632	61	pos	454300
71	Trans-Aconitate	C6H6O6	173.0082	0.8745	77.7	neg	433697
72	2'-O-Methyladenosine	C11H15N5O4	282.1193	1.5252	70.9	pos	426081
73	Beta-D-Glucosamine	C6H13NO5	180.0864	0.5966	60.1	pos	421377
74	Gentisic acid	C7H6O4	153.0182	3.1238	87.8	neg	406840
75	Gibberellin A53	C20H28O5	349.1979	10.6145	54.1	pos	396891
76	L-Phenylalanine	C9H11NO2	166.0862	1.8407	98.6	pos	391974
77	1,1-Dimethylpyrrolidinium-2-carboxylate	C7H13NO2	144.1018	0.6321	36.8	pos	383741
78	Methyl veratrate	C10H12O4	197.0807	3.1918	49	pos	378730
79	Guanosine	C10H13N5O5	282.0841	0.9909	92.9	neg	376159
80	6-Methyl-7-(3-oxobutyl)-bicyclo[4.1.0]heptan-3-one	C12H18O2	195.1378	7.42	79.7	pos	371022
81	Umbelliferone	C9H6O3	161.0234	3.9934	93.9	neg	358048
82	5,7-Dihydroxycoumarin	C9H6O4	177.0184	3.1238	60.6	neg	356214
83	Traumatic Acid	C12H20O4	227.1283	7.7413	98.1	neg	350777
84	Cinnamaldehyde	C9H8O	133.0648	2.6829	71.3	pos	345005
85	5-Hydroxymethyl-2-Furaldehyde	C6H6O3	127.0391	15.9819	91.5	pos	343315
86	Neocnidilide	C12H18O2	195.1378	12.2675	77.8	pos	343055
87	4-Guanidino-1-butanol	C5H13N3O	132.1131	0.7743	62.1	pos	336020

88	3,4'-Dihydroxypropiophenone	C9H10O3	167.0702	3.1918	72.3	pos	333650
89	Dihydromyricetin	C15H12O8	303.0495	5.0809	62.3	pos	322833
90	Aromadendrin	C15H12O6	287.056	5.5664	55.1	neg	319634
91	2-Phenylethyl formate	C9H10O2	151.0753	3.5536	74.8	pos	315107
92	1-Kestose	C18H32O16	503.1614	0.6268	96.7	neg	310454
93	Curcumenolactone C	C15H20O4	265.1431	2.8559	66.6	pos	304518
94	Ingol	C20H30O6	367.2083	7.2424	50.4	pos	303408
95	Benzoic acid	C7H6O2	123.0443	1.994	79.1	pos	298166
96	Apocynin	C9H10O3	167.0702	2.3418	71.2	pos	295539
97	3,4-Dihydroxybenzaldehyde	C7H6O3	137.0232	2.7513	68.3	neg	286770
98	D-Pantothenic acid	C9H17NO5	220.1178	2.3418	92.4	pos	284013
99	Isonicotinic acid	C6H5NO2	124.0394	0.7921	55.8	pos	279464
100	O-Cymol	C10H14	135.1168	3.7338	87.3	pos	278592
101	2-Methylbenzoic acid	C8H8O2	135.044	3.1446	48.9	neg	277383
102	Protocatechuic acid	C7H6O4	153.0183	2.1742	96.3	neg	276651
103	2-Pyrrolidinone	C4H7NO	86.0607	0.5788	75	pos	275770
104	(R)-(+)-Pulegone	C10H16O	153.1273	4.5794	83	pos	275336
105	Isotachioside	C13H18O8	301.0926	1.4578	39.1	neg	273849
106	DL-2-Aminoadipic acid	C6H11NO4	162.0759	0.543	53.3	pos	267378
107	Cnidilide	C12H18O2	195.1378	9.3585	96.4	pos	264981
108	Effusanin A	C20H28O5	349.198	7.4837	64.5	pos	264963
109	Cordycepin	C10H13N5O3	252.1085	0.9699	82.5	pos	264343
110	(-)-gamma-Cuparenol	C15H22O	273.1844	9.7954	54.2	pos	256640
111	Methylgingerol	C18H28O4	309.2054	9.7954	61.2	pos	255924
112	2-Hydroxy-4-methoxybenzaldehyde	C8H8O3	153.0545	3.1739	77.2	pos	248623
113	Estriol	C18H24O3	289.1794	10.3713	75.4	pos	241517
114	Momilactone B	C20H26O4	331.1874	9.2481	42.7	pos	240853
115	Gajutsulactone A	C15H22O2	235.1689	9.468	62.8	pos	237720
116	Cytidine	C9H13N3O5	244.0925	0.6144	73	pos	235061
117	Ligustilide	C12H14O2	191.1065	5.2332	75	pos	234105
118	Ectocarpene	C11H16	149.1324	8.3216	90.3	pos	232312
119	Quercetagetin	C15H10O8	317.0301	5.082	51.5	neg	229252
120	Isodemethylwedelolactone	C15H8O7	299.0196	5.5874	78.2	neg	216223
121	4-n-Hexylphenol	C12H18O	179.1428	13.1696	87.5	pos	212340
122	Furfural	C5H4O2	97.0289	0.6144	45.1	pos	211088
123	Benzyl acuminose	C18H26O10	401.145	3.3502	82.4	neg	210390
124	Methacrylamide	C4H7NO	86.0607	1.0411	48.9	pos	208746
125	Uridine	C9H12N2O6	243.0618	0.8745	96.6	neg	204812
126	Dehydroandrographolide	C20H28O4	333.203	9.5997	64.4	pos	202311
127	P-Coumaroyl quinic acid	C16H18O8	339.107	3.3586	91.4	pos	201622
128	3-O-Feruloylquinic acid	C17H20O9	367.1031	3.6189	97.4	neg	196940
129	Arctigenin	C21H24O6	373.1639	7.5923	60.2	pos	195482
130	Itaconic acid	C5H6O4	129.0181	0.6626	51	neg	193832
131	Eriodictyol chalcone	C15H12O6	287.0559	4.6856	87.1	neg	192084
132	6-Hydroxykaempferol	C15H10O7	301.0352	4.1613	55.8	neg	187412
133	Moracin P	C19H18O5	327.1221	7.2198	79	pos	185813

134	Plantagoside	C21H22O12	465.1033	2.8367	80.2	neg	184891
135	Thymidine	C10H14N2O5	241.0826	1.8401	51.4	neg	179079
136	Undecanedioic acid	C11H20O4	215.1282	7.0387	89.6	neg	175015
137	Dihydroactinidiolide	C11H16O2	181.1221	6.1897	82.7	pos	174651
138	Demethylwedelolactone	C15H8O7	299.0196	5.0616	88.9	neg	174164
139	Isovanillic acid	C8H8O4	169.0494	2.1289	88.9	pos	173695
140	Xylobiose	C10H18O9	281.0874	0.5729	49.9	neg	171706
141	Mulberroside A	C26H32O14	567.1713	2.7513	99.6	neg	171644
142	5-O-Caffeoylshikimic acid	C16H16O8	335.0771	3.4545	83.4	neg	166720
143	9-Octadecenamide	C18H35NO	282.2786	14.1067	97	pos	165341
144	Vitedoin A	C20H20O6	357.1326	6.2119	87.3	pos	164415
145	Trans-2-Octenal	C8H14O	125.096	4.7268	86.8	neg	161714
146	Skimmin	C15H16O8	325.0913	2.9475	97.9	pos	158699
147	Xanthoxylin	C10H12O4	197.0807	2.8742	77.2	pos	157169
148	Ibuprofen	C13H18O2	207.1378	3.456	87.2	pos	154952
149	5-O-Methylembelin	C18H28O4	307.1913	12.2818	66	neg	153420
150	Anemonin	C10H8O4	191.0341	4.0563	38.7	neg	149387
151	Trans-Zeatin	C10H13N5O	220.1178	2.1869	52.2	pos	148645
152	L-Leucine	C6H13NO2	173.1285	2.8559	41.7	pos	145637
153	5-Hydroxymaltol	C6H6O4	143.0339	1.6035	38.9	pos	145041
154	Apiopaeonoside	C20H28O12	459.1503	2.859	45.8	neg	144577
155	L-Asparagine	C4H8N2O3	131.045	0.5542	85.3	neg	142537
156	15-Hydroxydehydroabietic acid	C20H28O3	361.1993	12.755	41.5	neg	141325
157	Syringic acid	C9H10O5	199.06	2.4	94.8	pos	139385
158	Jioglutin E	C11H20O5	231.1233	4.5577	96.1	neg	138794
159	Valerophenone	C11H14O	163.1116	5.2332	67.8	pos	134577
160	Vanillic acid	C8H8O4	167.034	2.1337	98.3	neg	133719
161	Isosteviol	C20H30O3	363.215	13.055	43	neg	129743
162	4-Hydroxycinnamyl aldehyde	C9H8O2	149.0597	1.8598	83.5	pos	127106
163	Camphor	C10H16O	153.1272	0.3866	87.3	pos	123051
164	Suberic acid	C8H14O4	173.081	3.9729	87.3	neg	122175
165	Trans-Cinnamate	C9H8O2	149.0597	3.0606	71.1	pos	120779
166	Boschnialactone	C9H14O2	155.1065	7.4407	60.1	pos	120602
167	Alpha-Sinensal	C15H22O	219.1741	12.8602	66.2	pos	120593
168	Confertifoline	C15H22O2	235.1689	7.7241	84.5	pos	119932
169	Adenine	C5H5N5	134.046	1.2298	68.6	neg	119766
170	L-Hydroxyproline	C5H9NO3	114.0552	0.6321	43	pos	119063
171	2-Hydroxycinnamic acid	C9H8O3	165.0545	0.8988	77.9	pos	118105
172	2-keto-D-Gluconic acid	C6H10O7	193.0344	0.6086	57.1	neg	115050
173	Dihydrocarveyl acetate	C12H20O2	197.1534	4.8211	75.1	pos	114041
174	Quercetin 3-galactoside	C21H20O12	463.088	3.6189	48	neg	113974
175	Daphnetin	C9H6O4	177.0184	4.3669	64.3	neg	110439
176	(S)-5-Hydroxymethyl-2[5H]-furanone	C5H6O3	115.0392	2.8742	38.3	pos	109370
177	P-hydroxylphenylpropanol	C9H10O3	147.044	4.3258	40	neg	108694
178	Methyl linolenate	C19H32O2	293.2469	13.6923	62.9	pos	108544

179	(+/-)-Tryptophan	C11H12N2O2	205.097	2.5709	99.2	pos	107770
180	L-Aspartic Acid	C4H7NO4	132.029	0.5729	83.8	neg	107407
181	2,6-Dimethoxy-benzoic acid	C9H10O4	183.0651	2.457	56.5	pos	106949
182	Alantolactone	C15H20O2	233.1533	11.0332	81.9	pos	105984
183	Isobavachin	C20H20O4	323.1285	12.6481	95	neg	105130
184	Sesamin	C20H18O6	387.1431	6.6796	37.2	pos	104770
185	Arteannuin	C15H20O3	231.1376	0.3663	71.5	pos	102902
186	6-methyluracil	C5H6N2O2	127.0502	1.82	83.4	pos	102878
187	4-Methylumbelliferone	C10H8O3	177.0545	3.6337	94.4	pos	102361
188	Benzene-1,2,4-triol	C6H6O3	144.0655	1.3327	49.8	pos	101400
189	DL-Pipecolinic acid	C6H11NO2	147.1127	0.5074	85.1	pos	99178
190	Dictamnol	C12H18O	179.1429	4.8211	78.9	pos	99104
191	Tuberonic acid glucoside	C18H28O9	387.166	3.3912	52.4	neg	99077
192	5,7-Dihydroxychromone	C9H6O4	177.0184	3.5987	77.7	neg	97525
193	17-Hydroxyisolathyrol	C20H30O5	395.2048	9.2154	44.3	neg	94138
194	Paeonolide	C20H28O12	459.1505	2.4738	75.7	neg	93548
195	Sedanonic acid	C12H18O3	211.1326	9.2481	66.2	pos	92781
196	Osmanthuside H	C19H28O11	431.1555	3.7025	71.9	neg	88959
197	2-Hydroxycinnamaldehyde	C9H8O2	149.0597	1.2036	56.7	pos	87867
198	Inosine	C10H12N4O5	267.0723	1.0115	64.3	neg	84577
199	Pseudolaroside A	C13H16O8	299.077	1.6039	85.2	neg	82998
200	Luteolin	C15H10O6	285.0402	4.1613	83.5	neg	81897
201	D-Mannoheptulose	C7H14O7	209.0659	0.6086	82.1	neg	81699
202	Corchorifatty acid F	C18H32O5	311.221	7.42	85.2	pos	81179
203	Catalpol	C15H22O10	343.1034	2.5169	40.1	neg	78173
204	Hyperoside	C21H20O12	465.1023	3.6337	97.8	pos	76619
205	Nicotinamide	C6H6N2O	123.0554	0.7921	70.9	pos	76427
206	2-Phenylacetamide	C8H9NO	136.0756	0.8988	95.9	pos	76371
207	Caffeic Acid Methyl Ester	C10H10O4	195.0651	2.5896	69.2	pos	75089
208	Qing Hau Sau	C15H22O5	283.153	5.5654	94.2	pos	74976
209	Achillin	C15H18O3	247.1326	5.6322	44.9	pos	73355
210	Ethylbenzene	C8H10	107.0859	3.7338	89.5	pos	72961
211	Quercetin-7-O-beta-D-glucopyranoside	C21H20O12	463.0879	4.0148	88.8	neg	72650
212	1-O-Galloyl-beta-D-glucose	C13H16O10	331.0669	1.0688	56.3	neg	72545
213	Costunolide	C15H20O2	233.1534	2.7414	74.9	pos	71817
214	Methyl 4-hydroxybenzoate	C8H8O3	153.0546	3.8128	58.7	pos	70390
215	Caffeic acid 3-glucoside	C15H18O9	341.0877	2.4524	57.8	neg	69324
216	Isoferulic acid	C10H10O4	177.0545	4.6667	80.5	pos	68954
217	2,4,5,6-Tetrahydroxyphenanthrene	C14H10O4	243.065	4.2172	95	pos	68524
218	9-Oxononanoic acid	C9H16O3	171.1017	6.0948	90.9	neg	66086
219	5'-Prenyllicodione	C20H20O5	341.1377	11.8712	97.1	pos	65911
220	Jasmonic acid	C12H18O3	211.1326	12.617	98.2	pos	65037
221	Bergaptol I	C20H26O11	443.1589	2.0715	48.9	pos	64216
222	L-Isoleucine	C6H13NO2	132.102	3.0236	75.3	pos	63674
223	Oxyphyllenodiol B	C14H22O3	237.1491	10.1332	38.8	neg	63581

224	Phytosphingosine	C18H39NO3	318.2997	10.5713	95.2	pos	63357
225	Sinapyl alcohol	C11H14O4	211.0963	1.994	92.2	pos	63052
226	Grasshopper ketone	C13H20O3	225.1483	4.0156	76.4	pos	61708
227	5-Methoxysalicylic acid	C8H8O4	167.034	3.2055	97.6	neg	61097
228	Piceatannol	C14H12O4	245.0806	3.5324	85.7	pos	61035
229	Bicyclo[4.2.0]octa-1,3,5-triene	C8H8	105.0703	2.3619	95	pos	60927
230	Hydroquinone	C6H6O2	109.0282	2.1742	80.1	neg	60259
231	4-n-Pentylphenol	C11H16O	165.1273	6.6574	91	pos	60211
232	Ajugol	C15H24O9	313.1276	3.5536	51.4	pos	59950
233	4-hydroxy-benzoate	C7H6O3	137.0233	4.8735	92	neg	59827
234	Eseramine	C16H22N4O3	317.1604	4.5356	90.7	neg	58600
235	Gamma-Methoxyisoeugenol	C11H14O3	195.1015	3.3032	84.3	pos	57780
236	Parishin E	C19H24O13	459.114	2.7302	69.2	neg	56990
237	4-(beta-D-Glucopyranosyloxy)-3-methoxybenzoic acid	C14H18O9	348.1284	2.1289	63.9	pos	56203
238	Androsin	C15H20O8	327.1084	2.5379	80.9	neg	54237
239	Rosarin	C20H28O10	427.1605	4.664	82.2	neg	53176
240	Atractyloside A	C21H36O10	447.2234	5.987	93.6	neg	52202
241	DL-Glutamate	C5H9NO4	146.0447	0.5729	90.7	neg	52109
242	3,8-dihydroxy-urolithin	C13H8O4	273.0403	3.745	44.9	neg	51971
243	Crocusatin B	C10H16O3	183.1018	5.6924	35.2	neg	51371
244	Rosavin	C20H28O10	427.1604	4.3061	89.6	neg	51157
245	Estrone	C18H22O2	271.1689	10.6145	66.4	pos	51113
246	3-hydroxy-3-methyl-Glutaric acid	C6H10O5	323.0981	0.9712	50.2	neg	50691
247	Aesculetin	C9H6O4	177.0183	2.6673	92.1	neg	50305
248	Paeonoside	C15H20O8	327.1084	2.7725	93	neg	50138
249	Ferulaldehyde	C10H10O3	179.0702	4.6221	82	pos	49870
250	Piceatannol 3'-O-glucoside	C20H22O9	407.133	2.7792	92.1	pos	49160
251	Farnesyl acetone	C18H30O	280.263	13.8415	93.1	pos	48923
252	Shikimic acid	C7H10O5	173.0446	3.0219	66.4	neg	48646
253	Amurensin	C26H30O12	533.166	4.1825	42	neg	48512
254	Magnolone	C21H22O7	387.1431	7.0846	86.9	pos	48496
255	Guaiacol	C7H8O2	125.0598	3.1918	55.8	pos	48479
256	Nistose	C24H42O21	665.214	0.7963	98.4	neg	48195
257	Fa(7:0)	C7H14O2	189.1124	4.664	37.3	neg	47987
258	3-methylglutaric acid	C6H10O4	145.0495	2.4524	65.1	neg	47748
259	Vanillin	C8H8O3	151.039	3.8263	97.7	neg	46479
260	7-Methylcoumarin	C10H8O2	161.0596	1.8598	44.5	pos	46462
261	P-Mentha-1,3,8-triene	C10H14	135.1168	6.1897	72.4	pos	46188
262	Polydatin	C20H22O8	391.138	2.8921	88.2	pos	46118
263	Oxyresveratrol	C14H12O4	245.0806	2.7792	97.3	pos	45740
264	Isobavachalcone	C20H20O4	325.1428	12.6616	99.1	pos	45601
265	L-Histidine	C6H9N3O2	156.0766	2.3822	89.9	pos	45207
266	Hypoxanthine	C5H4N4O	137.0457	0.7921	49.5	pos	45060
267	Methyl 4-hydroxycinnamate	C10H10O3	177.0548	4.6005	42.3	neg	44935
268	Zingerone	C11H14O3	195.1015	3.5936	79.3	pos	44616

269	Scoparone	C11H10O4	207.0651	4.9495	97.5	pos	43966
270	(+)-Nootkatone	C15H22O	219.1741	3.9541	75.4	pos	43653
271	4-Hydroxybenzaldehyde	C7H6O2	123.0443	2.5335	83	pos	43556
272	Icosatrienoic acid	C20H34O2	324.2891	13.6282	63.2	pos	43404
273	Ornithine	C5H12N2O2	133.0971	0.4887	79.8	pos	43099
274	Oxyhydrastinine	C11H11NO3	206.0811	3.913	36.7	pos	42904
275	Chavicol	C9H10O	135.0804	3.0606	82.7	pos	42674
276	Beta-Hydroxypropiovanillone	C10H12O4	197.0808	2.5526	82.2	pos	42575
277	Calystegine B1	C7H13NO4	158.0811	2.1682	43.1	pos	42424
278	Eucommiol	C9H16O4	171.1014	12.1148	45.9	pos	42283
279	Leonuriside A	C14H20O9	333.1175	2.5709	63	pos	42077
280	(+)-Nortrachelogenin	C20H22O7	433.15	4.9362	57	neg	41841
281	Annuionone E	C13H22O3	225.1491	5.1025	74.3	neg	41706
282	Anethole	C10H12O	149.0961	3.7736	60.8	pos	41502
283	4-Isopropylbenzaldehyde	C10H12O	149.0961	3.456	81	pos	41176
284	Ferulic acid	C10H10O4	193.0498	3.4955	99.1	neg	40481
285	Gibberellin A24	C20H26O5	347.1823	8.1297	49.7	pos	40126
286	Gamma-Glu-Leu	C11H20N2O5	261.1442	2.7991	88	pos	39271
287	Astringin	C20H22O9	405.119	3.5365	94.7	neg	38132
288	6-Hydroxynicotinic acid	C6H5NO3	138.0185	0.9337	68.5	neg	38094
289	2,3-Dihydroxy-1-(4-hydroxy-3,5-dimethoxyphenyl)-1-propanone	C11H14O6	243.0861	1.7626	37.5	pos	37955
290	Farnesyl acetate	C17H28O2	265.2158	11.5828	78.9	pos	37792
291	Epomediol	C10H18O3	231.1233	4.9772	60.4	neg	37662
292	P-hydroxylbenzyl alcohol	C7H8O2	107.0495	2.9665	40.2	pos	37609
293	1-O-Feruloyl-beta-D-glucose	C16H20O9	355.1032	2.7513	69.1	neg	37524
294	L-Threonine	C4H9NO3	84.045	0.8632	39.5	pos	37421
295	Alpha-Cyclogeraniol acetate	C13H22O2	211.169	3.8723	82.3	pos	36597
296	Sesamolinol	C20H20O7	373.1275	4.1758	72.9	pos	36471
297	Estradiol	C18H24O2	273.1845	10.6145	50.4	pos	36181
298	Dihydroartemisinic acid	C15H24O2	237.1846	11.2549	47	pos	36100
299	2,6,6-Trimethyl-2-cyclohexene-1,4-dione	C9H12O2	153.0909	3.136	66	pos	35987
300	Fabiatrin	C21H26O13	487.1439	3.0792	86.5	pos	35622
301	Linderane	C15H16O4	261.1118	5.8553	57.2	pos	34974
302	3,5-Dimethoxy-4-hydroxybenzaldehyde	C9H10O4	181.0497	3.9934	90.8	neg	34561
303	(S)-tropic acid	C9H10O3	167.0701	0.2797	63.5	pos	33805
304	Rengyoside B	C14H24O8	319.1395	3.3083	76.8	neg	33693
305	Adipic acid	C6H10O4	145.0495	1.2711	35.9	neg	33112
306	Ilicic acid	C15H24O3	235.169	10.3488	59.3	pos	33078
307	Mulberrin	C25H26O6	423.1793	12.3109	95.6	pos	33022
308	2-Ethyltoluene	C9H12	121.1014	4.2172	88.5	pos	32602
309	Gomisin R	C22H24O7	401.1588	5.9002	89	pos	32515
310	Uracil	C4H4N2O2	113.0349	1.4275	64.5	pos	32329
311	Xanthatin	C15H18O3	247.1326	2.8559	69	pos	32307
312	2-hydroxyhexadecanoic acid	C16H32O3	271.2276	13.9441	98.3	neg	32303
313	Sanggenon H	C20H18O6	353.1027	10.944	45.7	neg	31823

314	Methyl orsellinate	C9H10O4	181.0498	2.0915	93.6	neg	31755
315	Pseudoionone	C13H20O	193.1586	4.5575	91.7	pos	31458
316	Geranic acid	C10H16O2	169.1222	3.713	55	pos	31405
317	Spiraeoside	C21H20O12	465.1023	4.0156	96.5	pos	31310
318	5-Acetylsalicylic acid	C9H8O4	181.0494	2.4	43.5	pos	31238
319	Sweroside	C16H22O9	357.1189	3.0412	81.8	neg	30882
320	Cuminyl acetate	C12H16O2	193.1222	6.6574	68.7	pos	30850
321	Tetralin	C10H12	133.1011	8.3216	94.3	pos	30781
322	D-Sorbitol	C6H14O6	181.0708	0.5729	91.9	neg	30536
323	Atractyloside G	C21H36O8	461.2393	3.9729	66.1	neg	30337
324	Pyrocatechol	C6H6O2	109.0282	1.375	47.4	neg	30288
325	3-(3-Hydroxyphenyl)propanoic acid	C9H10O3	165.0547	2.5379	78.4	neg	30029
326	C-veratroylglycol	C10H12O5	211.0606	2.4738	88.2	neg	30027
327	13-Apo-beta-carotenone	C18H26O	259.2053	12.7716	93.5	pos	30003
328	Panaxjapyne C	C17H26O3	279.1951	10.8116	60.6	pos	29549
329	3-Hydroxymorindone	C15H10O6	285.0403	5.6087	55.1	neg	29466
330	L-Arabinose	C5H10O5	149.0444	0.6086	92.5	neg	29338
331	Methyl-4-O-methylgallate	C9H10O5	197.0448	2.4091	92.9	neg	28895
332	Roughanic acid	C16H26O2	251.2002	12.2675	83.1	pos	28638
333	Acetophenone	C8H8O	121.065	3.713	55.1	pos	28490
334	Diglykokoll	C4H7NO4	134.0447	0.6144	46.2	pos	28089
335	Sucrose	C12H22O11	341.1086	15.8676	86.1	neg	27849
336	Alternariol	C14H10O5	257.0453	5.315	54.5	neg	27488
337	Alpha-Methylstyrene	C9H10	119.0856	12.093	88.8	pos	27234
338	1-Isopropyl-3-methylbenzene	C10H14	135.1167	8.3216	39.8	pos	26945
339	L-Tryptophan	C11H12N2O2	203.0819	2.5379	96.8	neg	26799
340	2-Octenoic acid	C8H14O2	143.1066	2.6456	41	pos	26542
341	Stemanthrene A	C17H18O4	287.125	5.6322	50	pos	26486
342	(-)-Glyceollin I	C20H18O5	337.108	6.1583	93.6	neg	26020
343	P-Cymene	C10H14	135.1167	12.7716	58.4	pos	25589
344	Picein	C14H18O7	297.0977	1.2089	65.4	neg	25577
345	Cleroindicin B	C8H14O3	203.0917	3.556	61	neg	25520
346	Hydroxytyrosol	C8H10O3	153.0545	2.0501	71	neg	24451
347	Linolenyl alcohol	C18H32O	282.2787	14.8418	85.3	pos	24213
348	Schizonepetoside E	C16H28O8	393.1765	3.9516	49.2	neg	24212
349	Dibutyl phthalate	C16H22O4	279.1586	13.3703	61.9	pos	23999
350	Salicylaldehyde	C7H6O2	123.0442	0.8988	62.3	pos	23899
351	4-Methylcatechol	C7H8O2	125.0599	2.1289	71	pos	23655
352	Geranylacetone	C13H22O	195.1742	4.2172	84.3	pos	23622
353	Oxaloacetate	C4H4O5	130.9973	0.8162	41.8	neg	23620
354	Albuterol	C13H21NO3	240.1591	5.7448	74.9	pos	23545
355	Dihydroartemisinin	C15H24O5	283.1549	7.4107	65.9	neg	23524
356	Ingenol	C20H28O5	393.189	7.4552	41.1	neg	23318
357	Isopeonol	C9H10O3	165.0547	3.2676	62.9	neg	23227
358	Curcumol	C15H24O2	237.1847	3.9541	87.3	pos	23132

359	Feruloylputrescine	C14H20N2O3	263.1399	3.2055	86.8	neg	23132
360	Cis-Resveratrol	C14H12O3	229.0857	2.8921	92	pos	23062
361	Nicotianamine	C12H21N3O6	302.1356	1.6679	64.4	neg	23048
362	Sesamol	C7H6O3	121.0286	2.9288	54.8	pos	23013
363	3,5,7,2',6'-Pentahydroxyflavanone	C15H12O7	305.0651	2.8364	87	pos	22920
364	Capillanol	C12H14O	175.1118	6.6574	73.9	pos	22790
365	3-Hydroxy-2-Methylpyridine	C6H7NO	110.0604	15.8934	39.1	pos	22542
366	Sedanonic acid lactone	C12H16O2	193.1221	12.2675	79.6	pos	22430
367	Butin	C15H12O5	271.061	4.7064	57.1	neg	22346
368	Eremophila-1(10),8,11-triene	C15H22	203.1792	13.1037	86.2	pos	22310
369	7,8-Dihydroxyflavone	C15H10O4	287.091	8.282	37.1	pos	22038
370	Melazolide A	C11H16O4	195.1015	5.0378	47.1	pos	21700
371	2-Methoxy-4-vinylphenol	C9H10O2	149.0597	2.2806	87.2	neg	21540
372	Aristolone	C15H22O	201.1636	4.6885	37.7	pos	21514
373	Methyl cinnamate	C10H10O2	163.0751	2.8559	65.2	pos	21493
374	Dehydrolindestrenolide	C15H16O2	229.1223	5.6102	70.7	pos	21306
375	3,4-Bis(methoxycarbonyl)benzoic acid	C11H10O6	237.04	3.9516	90.3	neg	21198
376	Herniarin	C10H8O3	177.0545	3.0792	84.8	pos	20866
377	Glutarate	C5H8O4	133.0495	1.9745	47.2	pos	20831
378	3-Cyano-L-alanine	C4H6N2O2	113.0343	0.5542	41.1	neg	20664
379	Pimelic acid	C7H12O4	159.0652	3.2055	88.2	neg	20642
380	Indole-3-aldehyde	C9H7NO	146.06	2.5709	95	pos	20367
381	Isovanillin	C8H8O3	151.039	2.5169	77.3	neg	20203
382	Andrographolide	C20H30O5	395.2049	11.0555	46	neg	20143
383	Rishitin	C14H22O2	223.1689	13.0367	89.9	pos	19913
384	Longicamphenylone	C14H22O	207.1741	11.2549	82.5	pos	19769
385	Isorhamnetin	C16H12O7	315.051	5.8589	90.6	neg	18860
386	(-)-Abscisic acid	C15H20O4	263.1287	5.5874	96.3	neg	18608
387	Inositol	C6H12O6	225.0609	0.5542	81.3	neg	18361
388	Melilotoside	C15H18O8	325.0928	2.5169	83.2	neg	18302
389	L-Tyrosine	C9H11NO3	182.081	0.6677	95	pos	18186
390	Atractylenolide II	C15H20O2	233.1532	12.5281	96.9	pos	18010
391	Perillene	C10H14O	151.1116	12.2675	88.2	pos	17696
392	Xylitol	C5H12O5	151.06	0.5907	85.2	neg	17597
393	Crocusatin G	C12H20O2	195.1382	8.8848	47.3	neg	17564
394	(-)-Carvone	C10H14O	151.1117	4.5794	83.3	pos	17339
395	Trans-3-Hydroxycinnamate	C9H8O3	165.0545	0.2797	53.6	pos	17327
396	Sphinganine	C18H39NO2	302.3048	12.2244	59.2	pos	17211
397	L-Valine	C5H11NO2	118.0865	15.3966	68.8	pos	17117
398	4-Methoxysalicylic acid	C8H8O4	151.0389	2.9288	39.8	pos	17116
399	Shinjulactone C	C20H22O7	375.1432	4.7097	46.7	pos	17109
400	L-Noradrenaline	C8H11NO3	170.0811	2.2068	54.3	pos	16873
401	Esculin	C15H16O9	339.0719	2.1543	94.9	neg	16819
402	Liquiritigenin	C15H12O4	255.066	7.7413	73.1	neg	16672
403	Nonanoic acid	C9H18O2	159.1378	5.0157	39.9	pos	16581

404	Artemisinic acid	C15H22O2	235.169	3.8319	81.1	pos	16476
405	Sorbic acid	C6H8O2	113.06	2.5896	63.6	pos	16379
406	Cadalene	C15H18	199.1479	3.8319	62.7	pos	16226
407	Sphingosine	C18H37NO2	300.2892	12.4194	87.6	pos	16049
408	Chrysophanol	C15H10O4	287.091	5.4096	39.6	pos	16009
409	(+)-Anwulignan	C20H24O4	373.1628	10.5966	56.3	neg	15980
410	Isokobusone	C14H22O2	223.169	9.2036	53.5	pos	15874
411	L-Ribose	C5H10O5	149.0444	3.1446	47.9	neg	15733
412	Nortricycloekasantalic acid	C11H16O2	181.1221	11.5828	67.8	pos	15690
413	Ethyl 4-methoxycinnamate	C12H14O3	207.1014	4.3195	66.8	pos	15125
414	Acetosyringone	C10H12O4	195.0653	3.9729	36.2	neg	14962
415	Emodin	C15H10O5	271.0596	8.282	44.4	pos	14447
416	8-Formyl-5-O-methylophiopogonanone B	C20H20O6	355.1184	9.781	42.9	neg	14389
417	4-acetoxyphenol	C8H8O3	151.039	3.0219	59.1	neg	14247
418	Diisobutyl phthalate	C16H22O4	279.1568	8.9125	39.3	pos	14113
419	Kaempferol	C15H10O6	285.0403	6.7524	76.4	neg	13980
420	L-(-)-3-Phenyllactic acid	C9H10O3	165.0547	4.0351	85.8	neg	13900
421	Indene	C9H8	117.0701	4.3195	92.5	pos	13825
422	Purpurin	C14H8O5	255.0296	4.3061	87.9	neg	13807
423	(2R)-2-(3,4-Dihydroxyphenyl)-3,5,7-trihydroxy-2,3-dihydrochromen-4-one	C15H12O7	303.0508	2.8367	98.3	neg	13766
424	Micheliolide	C15H20O3	249.1482	10.9889	35.9	pos	13721
425	Licoagroside D	C22H24O10	493.135	5.4177	76.4	neg	13651
426	Morin	C15H10O7	303.0493	11.8281	86.1	pos	13591
427	4-Acetyl-1-methylcyclohexene	C9H14O	139.1117	4.9066	64.9	pos	13403
428	Kobusone	C14H22O2	223.169	10.3928	68.8	pos	13231
429	Veratric acid	C9H10O4	183.065	15.9376	38.8	pos	13190
430	Scytalone	C10H10O4	177.0545	2.7603	70.4	pos	13093
431	Ricinoleic acid	C18H34O3	297.243	13.36	53	neg	12896
432	(+)-7-epi-Syringaresinol 4'-glucoside	C28H36O13	579.2078	4.4717	97.8	neg	12557
433	Jasmone	C11H16O	165.1272	11.8933	73.4	pos	12550
434	Lupinalbin A	C15H8O6	317.0652	5.8777	81.9	pos	12474
435	Cnidioside A	C17H20O9	367.1032	3.0822	41.6	neg	12358
436	Eriodictyol	C15H12O6	289.0703	4.4691	76.6	pos	12247
437	Licoleafol	C20H20O7	371.1134	5.6496	43.7	neg	11930
438	Butein	C15H12O5	273.0754	4.7317	55	pos	11913
439	(-)-Fenchone	C10H16O	135.1168	7.3538	45.8	pos	11838
440	Blumenol B	C13H22O3	227.1638	11.2549	67.8	pos	11762
441	Emodin anthrone	C15H12O4	255.066	8.8848	82.9	neg	11655
442	Nepodin	C13H12O3	215.0707	9.9572	46.3	neg	11631
443	Xestodecalactone A	C14H16O5	265.1067	3.7542	39.6	pos	11589
444	Benzofuran	C8H6O	119.0494	3.3773	82.5	pos	11359
445	3-Methylbenzaldehyde	C8H8O	121.065	2.457	62.5	pos	11331
446	Terbutaline	C12H19NO3	226.1435	4.3195	37.4	pos	11264
447	Ginkgolic acid	C20H32O3	365.2327	12.4968	37.7	neg	11223
448	Senkyunolide H	C12H16O4	225.1118	7.3094	75.3	pos	11150

449	9,10-Dihydroxystearate	C18H36O4	315.2538	12.7763	90.2	neg	11109
450	[8]-Shogaol	C19H28O3	305.2107	13.2584	61.6	pos	11066
451	Myristoleic acid	C14H26O2	285.207	11.1876	54.3	neg	11004
452	Kuwanon G	C40H36O11	691.218	11.7841	94.4	neg	10863
453	2,6-Dihydroxypurine	C5H4N4O2	151.025	0.8365	85.3	neg	10687
454	Sinapyl aldehyde	C11H12O4	207.0656	4.6221	95.4	neg	10568
455	Abietic acid	C20H30O2	320.258	11.0771	35.2	pos	10565
456	Isochlorogenic acid C	C25H24O12	515.1191	4.5356	97.4	neg	10443
457	Palmitic amide	C16H33NO	256.263	14.0499	40.6	pos	10393
458	D-Cathine	C9H13NO	152.1069	9.8385	81.5	pos	10281
459	Indole	C8H7N	118.0654	2.6278	67.5	pos	10133
460	Liquiritin	C21H22O9	417.119	6.2459	75.8	neg	10084
461	3,4-Dimethylstyrene	C10H12	133.1011	12.2675	66.8	pos	10035
462	Arachidonic Acid (peroxide free)	C20H32O2	305.2469	13.5681	37.9	pos	9884
463	Licoflavonol	C20H18O6	355.1169	10.9443	88.5	pos	9866
464	5,7-Dihydroxyisoflavone	C15H10O4	255.0647	5.2771	65.5	pos	9813
465	N,N'-Dicyclohexylurea	C13H24N2O	225.1958	9.2922	51.3	pos	9657
466	Isosinensetin	C20H20O7	355.1169	11.6943	45	pos	9369
467	Carnosol	C20H26O4	375.1787	12.2818	67.1	neg	9362
468	Kushenol F	C25H28O6	407.1845	13.1696	51	pos	9329
469	2-Adamantanone	C10H14O	151.1116	11.8933	53.5	pos	9174
470	2-Chromanone	C9H8O2	147.044	2.7725	50.5	neg	9063
471	7-Hydroxycadalene	C15H18O	215.1427	2.7414	37.1	pos	9032
472	Pinoresinol diglucoside	C32H42O16	700.2804	3.6137	66.5	pos	8996
473	3-Methyl-2-oxobutanoic acid	C5H8O3	117.0549	0.2797	46.2	pos	8921
474	Scopolamine hydrobromide	C17H21NO4	304.154	3.0606	90.6	pos	8918
475	Isoliquiritigenin	C15H12O4	257.0805	3.6137	68.9	pos	8740
476	4-Ethylphenol	C8H10O	123.0807	3.6543	67.8	pos	8673
477	Germacrone	C15H22O	219.174	13.3254	91.7	pos	8563
478	Gamma-GLU-PHE	C14H18N2O5	295.1286	3.004	82.5	pos	8509
479	Senkyunolide	C12H16O2	193.1222	12.8818	52.5	pos	8507
480	Linolenic acid ethyl ester	C20H34O2	307.2627	13.8789	69.2	pos	8456
481	Methyl geranate	C11H18O2	183.1379	4.4915	40.5	pos	8372
482	Purpurogallin	C11H8O5	219.0292	2.7097	67.8	neg	8291
483	M-Xylene	C8H10	107.0859	8.3216	75.6	pos	8163
484	Gingerol	C17H26O4	293.1757	10.7034	94.3	neg	8131
485	Santene	C9H14	123.1169	12.4194	80.1	pos	8065
486	Dihydrojasmonic acid, methyl ester	C13H22O3	227.1638	13.236	56.8	pos	8003
487	H-ile-ile-oh	C12H24N2O3	243.171	3.0412	62.1	neg	8002
488	Atractylenolide III	C15H20O3	231.1377	10.8341	94.2	pos	7834
489	10-hydroxy-2E-decenoic acid	C10H18O3	187.1327	5.9002	78.3	pos	7800
490	Dihydronepetalactone	C10H16O2	169.1222	5.9002	53	pos	7699
491	5,7,3'-Trihydroxy-4'-methoxyflavanone	C16H14O6	301.0716	6.8189	36.2	neg	7641
492	Caryophyllene epoxide	C15H24O	221.1897	11.3414	86.5	pos	7560
493	P-Tolualdehyde	C8H8O	121.0649	10.6361	80.4	pos	7446

494	Isofraxidin	C11H10O5	223.0598	4.4915	95.6	pos	7357
495	Carnitine	C7H15NO3	162.1123	15.3966	38.3	pos	7119
496	2,6-Dimethylnaphthalene	C12H12	157.1011	6.6574	58.1	pos	7007
497	3-Ethyltoluene	C9H12	121.1013	8.3216	80.5	pos	6979
498	3,5-Dicaffeoylquinic acid	C25H24O12	515.119	4.4083	94.9	neg	6915
499	Alpha-Linolenic acid	C18H30O2	277.2171	13.0764	67.6	neg	6818
500	Citrulline	C6H13N3O3	174.0873	0.5729	70.8	neg	6800
501	Sinapic acid	C11H12O5	223.0606	3.8469	97.7	neg	6761
502	4-Pyridinol	C5H5NO	96.0449	15.9156	38.6	pos	6671
503	O-Xylene	C8H10	107.0859	7.42	67.6	pos	6478
504	(R)-ar-Turmerone	C15H20O	217.1585	12.7273	39.6	pos	6208
505	Pentadecanal	C15H30O	271.2277	12.861	37.3	neg	6151
506	5-Methyl-2-furaldehyde	C6H6O2	111.0444	5.2332	69.7	pos	6098
507	(-)-Salsolinol	C10H13NO2	162.0912	4.1375	72.4	pos	5892
508	Quercetin	C15H10O7	303.0493	12.7273	84.1	pos	5815
509	Alpha,4-Dimethylstyrene	C10H12	133.1012	5.4096	89.8	pos	5746
510	Moracin N	C19H18O4	311.1274	10.527	85.4	pos	5680
511	Furanodienon	C15H18O2	231.1376	9.0919	70.7	pos	5666
512	Acetyleugenol	C12H14O3	207.1014	7.3316	55.4	pos	5659
513	Thymol	C10H14O	133.1012	7.42	40.6	pos	5646
514	Rubescensin A	C20H28O6	363.1808	8.1154	46.2	neg	5626
515	4,2',4',alpha-Tetrahydroxydihydrochalcone	C15H14O5	257.0804	7.8137	40.5	pos	5399
516	Pelletierine	C8H15NO	142.1226	3.7338	70.3	pos	5378
517	2'-Deoxymugineic acid	C12H20N2O7	305.1338	0.6321	42.1	pos	5366
518	Gallic acid	C7H6O5	169.0133	1.1276	95.5	neg	5359
519	Verbenone	C10H14O	151.1116	10.6145	43.3	pos	5358
520	Brosimacutin D	C20H20O5	341.1378	10.0138	77.4	pos	5189
521	1-Methoxynaphthalene	C11H10O	159.0803	10.6145	76.3	pos	5144
522	Curcumenol	C15H22O2	235.1689	3.3952	83.2	pos	4859
523	Alpha-Calacorene	C15H20	201.1635	12.926	68.1	pos	4764
524	(+)-alpha-Curcumene	C15H22	203.1792	14.6719	89	pos	4755
525	6-Hydroxyrubiadin	C15H10O5	269.0452	8.2477	64.5	neg	4671
526	(S)-(+)-alpha-Phellandrene	C10H16	137.1324	13.0367	78.4	pos	4593
527	Palmitoyl Ethanolamide	C18H37NO2	300.2893	13.8897	57.9	pos	4592
528	Lumichrome	C12H10N4O2	243.0874	4.6667	92.8	pos	4547
529	Parthenolide	C15H20O3	231.1377	13.0815	79.4	pos	4505
530	Dillapiole	C12H14O4	221.0813	8.6854	42.9	neg	4497
531	Pterosin G	C14H18O3	235.1326	6.1678	73.1	pos	4378
532	Curcolone	C15H18O3	291.1236	10.1996	39.1	neg	4351
533	Parthenin	C15H18O4	263.1268	8.8236	60.9	pos	4350
534	Isoeugenol	C10H12O2	163.0754	6.1152	86.9	neg	4309
535	Myristic acid	C14H28O2	246.2424	7.7687	52.5	pos	4280
536	Beta-Mangostin	C25H28O6	425.1956	12.617	56.8	pos	4253
537	2,3-Butanediol	C4H10O2	73.0655	1.0769	58.4	pos	4133
538	Euparin	C13H12O3	215.0707	10.3987	44.2	neg	4117

539	5,7,4'-Trihydroxy-8-methylflavanone	C16H14O5	269.0805	12.6616	73.4	pos	4052
540	13Z-Docosenamide	C22H43NO	338.341	15.2388	77.2	pos	4039
541	(S)-(-)-Perillyl alcohol	C10H16O	135.1167	13.1696	93.2	pos	4001
542	(6E)-8-Oxogeraniol	C10H16O2	169.1221	12.9479	67.2	pos	3998
543	Fraxinol	C11H10O5	223.0598	4.1962	76.5	pos	3834
544	Dehydrocostus lactone	C15H18O2	231.1377	14.433	84.5	pos	3754
545	Hexadecanedioic acid	C16H30O4	285.2069	12.6263	89.6	neg	3557
546	Delta-Hexalactone	C6H10O2	115.0756	9.1817	55.9	pos	3548
547	Turmeronol B	C15H20O2	233.1534	3.913	46.3	pos	3404
548	Pteroside A	C21H30O8	411.2005	4.278	62.5	pos	3388
549	6-Methyl indole	C9H9N	132.0808	2.5709	85.5	pos	3274
550	Damnacanthol	C16H12O5	285.0753	11.8712	72.5	pos	3241
551	4,7-Dimethyl-1-tetralone	C12H14O	175.1118	3.8319	63.5	pos	2891
552	4-Hydroxycinnamamide	C9H9NO2	181.097	0.9521	36.3	pos	2885
553	Licoflavone C	C20H18O5	337.1079	10.7469	65.9	neg	2818
554	Kuwanon A	C25H24O6	421.1637	12.8602	94.4	pos	2771
555	Methylhydroquinone	C7H8O2	125.0598	6.1897	65.9	pos	2754
556	(-)-Syringaresinol di-O-glucoside	C34H46O18	787.2654	3.745	96.8	neg	2741
557	Soyasapogenol C	C30H48O2	423.3611	14.208	38.4	pos	2731
558	4-Methoxycinnamic acid	C10H10O3	161.0596	2.7792	68	pos	2521
559	3-Butylidenephthalide	C12H12O2	189.0909	6.9942	68.5	pos	2498
560	Moracin D	C19H16O4	309.1118	11.2549	73.8	pos	2236
561	Chamazulene	C14H16	185.1323	13.1037	87.8	pos	2198
562	Indoleacetic acid	C10H9NO2	176.0705	4.0769	68.3	pos	2171
563	6-Paradol	C17H26O3	261.1846	9.2922	45.9	pos	2163
564	Narchinol A	C12H14O3	207.1014	8.8236	63.4	pos	2158
565	Piperine	C17H19NO3	286.1434	10.8341	96	pos	2134
566	Brazilin	C16H14O5	287.0911	2.7792	67.5	pos	2116
567	5,7-Dihydroxyflavone	C15H10O4	253.0504	6.2682	70.1	neg	2028
568	Arglabin	C15H18O3	247.1325	10.7902	75.4	pos	2011
569	2-buten-1-one, 1-(2,6,6-trimethyl-1,3-cyclohexadien-1-yl)-	C13H18O	191.1429	2.7414	77.5	pos	1949
570	Licochalcone B	C16H14O5	285.0766	9.2593	72.9	neg	1784
571	4-Methyl-5-thiazoleethanol	C6H9NOS	144.0477	1.5252	53.4	pos	1771
572	Methylnissolin	C17H16O5	301.1065	9.9919	43.4	pos	1758
573	Vulgarin	C15H20O4	265.1431	7.8593	63.5	pos	1719
574	Curdione	C15H24O2	237.1845	6.9489	78.3	pos	1669
575	Dehydro-1,8-cineole	C10H16O	151.1118	6.8623	82.6	neg	1610
576	Anthracene	C14H10	179.0853	12.6392	93.1	pos	1533
577	Apigenin	C15H10O5	269.0454	6.5531	79.5	neg	1526
578	Stemofuran A	C14H10O3	227.0699	2.7792	64.8	pos	1495
579	Nardosinone	C15H22O3	215.1428	13.5521	36.1	pos	1482
580	Incensole	C20H34O2	307.2625	13.4839	49.3	pos	1459
581	Safflomin A	C27H32O16	630.2021	3.5138	40.2	pos	1445
582	1F-fructofuranosylnystose	C30H52O26	827.2667	0.7963	95	neg	1383
583	1,4-Dicaffeoylquinic acid	C25H24O12	499.1227	4.3608	73.7	pos	1372

584	6-beta-D-Glucopyranosyl-8-beta-D-ribopyranosylapigenin	C26H28O14	563.1402	3.556	93	neg	1330
585	Styrene	C8H8	105.0702	12.5281	97.6	pos	1313
586	Epinepetalactone	C10H14O2	167.1065	11.122	67.2	pos	1309
587	Isoalantolactone	C15H20O2	233.1533	14.0499	90.7	pos	1293
588	6-Methylcoumarin	C10H8O2	161.0596	7.9945	64.6	pos	1261
589	Pc(16:0/0:0)	C24H50NO7P	496.339	13.2807	97.1	pos	1190
590	Wighteone	C20H18O5	337.1079	11.6966	55.3	neg	1119
591	Dehydroabietic acid	C20H28O2	301.2157	12.071	70.8	pos	1108
592	Chrysoeriol	C16H12O6	299.0558	6.951	98.2	neg	1037
593	Morusin	C25H24O6	421.1634	13.192	97.7	pos	932
594	Beta-Calacorene	C15H20	201.1635	13.6139	85.3	pos	858
595	7-Isopropyl-1,4-dimethylazulene	C15H18	199.1479	12.071	87	pos	822
596	(2R)-and (2S)-eriodictyol-7-O-beta-D-glucopyranoside	C21H22O11	451.1229	3.5138	85.1	pos	813
597	Pheophorbide a	C35H36N4O5	593.2749	14.1462	81.3	pos	799
598	Actidione	C15H23NO4	264.159	6.7683	76	pos	794
599	Forskolin	C22H34O7	411.2358	6.9715	36.5	pos	775
600	H-Glu-Tyr-OH	C14H18N2O6	291.0986	2.6464	52.8	neg	743
601	Lycoperodine 1	C12H12N2O2	215.082	3.1446	90.7	neg	724
602	Alpha-Corocalene	C15H20	201.1635	13.3254	82.6	pos	713
603	Trans,trans-2,4-Heptadienal	C7H10O	111.0808	11.5173	60.6	pos	711
604	Oxypeucedanin hydrate	C16H16O6	305.1016	5.7222	97.8	pos	701
605	Coniferyl alcohol	C10H12O3	163.0752	10.8341	40	pos	667
606	Daidzein	C15H10O4	255.0647	11.8281	82.2	pos	610
607	Norharman	C11H8N2	169.076	3.4744	52.3	pos	573
608	Gamma-Calacorene	C15H20	201.1635	13.8217	93.4	pos	515
609	Kainic acid	C10H15NO4	212.0922	3.8059	57.4	neg	504
610	1,3-O-Dicaffeoylquinic acid	C25H24O12	515.1198	3.4335	96.8	neg	497
611	3,3',5-Trimethoxybibenzyl	C17H20O3	331.1546	13.4258	41.8	neg	402
612	Ponicidin/Rubescensin B	C20H26O6	363.1773	4.4467	64.7	pos	396
613	Byakangelicol	C17H16O6	317.1015	5.9673	91.4	pos	392
614	Alpha-Farnesene	C15H24	205.1948	13.1696	62	pos	373
615	Atropine	C17H23NO3	290.1747	3.6337	86.9	pos	363
616	Tetrahydroberberine	C20H21NO4	372.1776	10.0585	35.4	pos	352
617	Eriodictyol-7-O-glucoside	C21H22O11	451.123	4.1758	82.4	pos	294
618	Phenethyl caffeate	C17H16O4	283.0973	10.6172	81.3	neg	293
619	Dihydrooroxylin	C16H14O5	285.0767	8.2477	65.5	neg	290
620	Homoorientin	C21H20O11	447.0931	3.5987	87.8	neg	276
621	Longifolenaldehyde	C15H24O	221.1898	9.8167	83.7	pos	224
622	Glabrone	C20H16O5	335.0923	11.5407	53.5	neg	218
623	Dehydrodiisoeugenol	C20H22O4	359.1844	12.6842	43.4	pos	211
624	7-Demethylsuberosin	C14H14O3	229.0864	10.3987	90.7	neg	197
625	Androstenedione	C19H26O2	328.2265	13.4611	35.1	pos	169
626	Pterosin Z	C15H20O2	274.1796	12.5281	78.4	pos	152
627	Tryptamine	C10H12N2	161.1073	2.9288	94	pos	141
628	Aristolene	C15H24	205.1949	12.9696	79.5	pos	134

629	Acenaphthene	C12H10	155.0853	12.6392	91.2	pos	125
630	(+)-longifolene	C15H24	205.195	5.9898	70.8	pos	97
631	Vitexin	C21H20O10	431.098	3.9729	89.2	neg	81
632	Atractylenolide I	C15H18O2	248.1642	8.8012	84.7	pos	76
633	Atractylodin	C13H10O	182.0724	12.6392	45.6	pos	73
634	Benzyl benzoate	C14H12O2	213.0907	12.5281	71.7	pos	70
635	Pterosin B	C14H18O2	183.1166	13.1696	51.8	pos	62
636	5,6-Dehydrokawain	C14H12O3	227.0708	10.944	46.3	neg	54
637	Aurantiamide acetate	C27H28N2O4	445.2113	11.6943	86.6	pos	43
638	Atalantoflavone	C20H16O5	335.0922	12.5833	82.9	neg	42
639	1-O-Acetylbritannilactone	C17H24O5	291.1585	13.3481	73.4	pos	40
640	Trans-Stilbene	C14H12	181.1009	13.1264	81.4	pos	33
641	N-Phenyl-1-naphthylamine	C16H13N	220.1117	12.6392	49.2	pos	26
642	Beta-Eudesmol	C15H26O	264.2318	13.3027	86.5	pos	23
643	Linderalactone	C15H16O3	245.117	8.8236	57.8	pos	23
644	Pterostilbene	C16H16O3	257.1167	12.6392	36	pos	16
645	Diphenylcyclopropenone	C15H10O	239.1062	13.2807	42	pos	4
